# Surface Structure of Alkyl/Fluoroalkylimidazolium Ionic–Liquid Mixtures

**DOI:** 10.1021/acs.jpcb.1c10460

**Published:** 2022-02-28

**Authors:** Simon M. Purcell, Paul D. Lane, Lucía D’Andrea, Naomi S. Elstone, Duncan W. Bruce, John M. Slattery, Eric J. Smoll, Stuart J. Greaves, Matthew L. Costen, Timothy K. Minton, Kenneth G. McKendrick

**Affiliations:** † Institute of Chemical Sciences, School of Engineering and Physical Sciences, Heriot-Watt University, Edinburgh EH14 4AS, U.K.; ‡ Department of Chemistry and Biochemistry, Montana State University, Bozeman, Montana 59717, United States; § Ann and H.J. Smead Department of Aerospace Engineering Sciences, University of Colorado Boulder, Boulder, Colorado 80303, United States; ∥ Department of Chemistry, University of York, Heslington, York YO10 5DD, U.K.

## Abstract

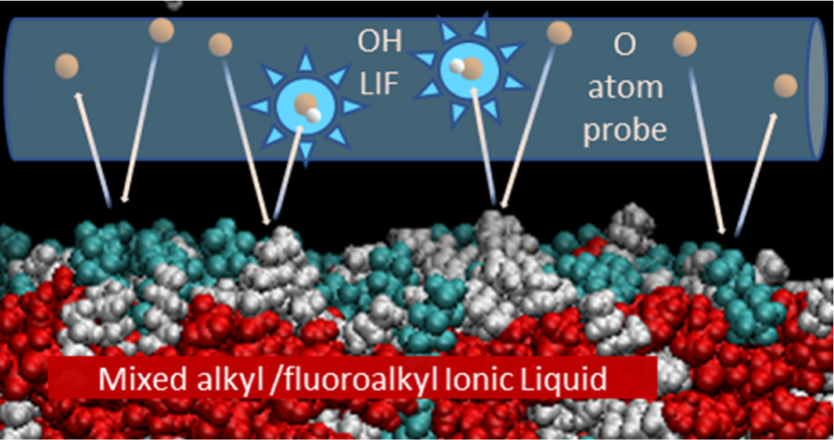

The gas–liquid interface of ionic liquids (ILs) is critically important in many applications, for example, in supported IL phase (SILP) catalysis. Methods to investigate the interfacial structure in these systems will allow their performance to be improved in a rational way. In this study, reactive-atom scattering (RAS), surface tension measurements, and molecular dynamics (MD) simulations were used to study the vacuum interface of mixtures of partially fluorinated and normal alkyl ILs. The underlying aim was to understand whether fluorinated IL ions could be used as additives to modify the surface structure of one of the most widely used families of alkyl ILs. The series of ILs 1-alkyl-3-methylimidazolium bis(trifluoromethylsulfonyl)imide ([C_*n*_mim][Tf_2_N]) with *n* = 4–12 were mixed with a fixed-length, semiperfluorinated analogue (1*H*,1*H*,2*H*,2*H*-perfluorooctyl)-3-methylimidazolium bis(trifluoromethylsulfonyl)imide ([C_8_mimF_13_][Tf_2_N]), forming [C_*n*_mim]_(1–*x*)_[C_8_mimF_13_]_*x*_[Tf_2_N] mixtures, where *x* is the bulk mole fraction of the fluorinated component. The RAS-LIF method combined O-atom projectiles with laser-induced fluorescence (LIF) detection of the product OH as a measure of surface exposure of the alkyl chains. For [C_8_mim]_(1–*x*)_[C_8_mimF_13_]_*x*_[Tf_2_N] mixtures, RAS-LIF OH yields are below those expected from stoichiometry. There are quantitatively consistent negative deviations from linearity of the surface tension. Both results imply that the lower-surface-tension fluoroalkyl material dominates the surface. A similar deficit is found for alkyl chain lengths *n* = 4, 6, 8, and 12 and for all (nonzero) *x* investigated by RAS-LIF. Accessible-surface-area (ASA) analyses of the MD simulations for [C_*n*_mim]_(1–*x*)_[C_8_mimF_13_]_*x*_[Tf_2_N] mixtures qualitatively reproduce the same primary effect of fluoro-chain predominance of the surface over most of the range of *n*. However, there are significant quantitative discrepancies between MD ASA predictions and experiment relating to the strength of any *n*-dependence of the relative alkyl coverage at fixed *x*, and on the *x*-dependence at fixed *n*. These discrepancies are discussed in the context of detailed examinations of the surface structures predicted in the MD simulations. Potential explanations, beyond experimental artifacts, include inadequacies in the classical force fields used in the MD simulations or the inability of simple ASA algorithms to capture dynamical factors that influence RAS-LIF yields.

## Introduction

Ionic liquids (ILs) are regarded as salts that are liquid below 100 °C. They are typically composed of large organic cations paired with a wide variety of anions, with the absence of molecular symmetry helping to disrupt efficient packing and delocalized charges minimizing the strength of Coulomb interactions. ILs have attracted a very large amount of interest in recent decades due to their prospects as alternative reaction media, with diverse potential applications spanning, for example, catalysis,^1−10^ carbon-capture and storage,^11,12^ biomass processing,^13,14^ electrolytes in batteries,^15−20^ supercapacitors,^21,22^ and dye-sensitized solar cells.^23^

The focus here will be on the surface properties of ILs, which have in general been recognized to be of substantial interest because they are expected to play a crucial role in a number of very important applications; these include gas separation, sequestration and forms of multiphase catalysis (e.g., supported ionic-liquid phase catalysis^24−29^). In particular, we will address the question of whether adding a second component (in this case a fluorinated IL) in known proportions might provide a method to systematically alter the surface properties of one of the most widely used alkyl IL families. This has interesting potential prospects for future exploitation. For example, in multiphase catalytic systems, tailored layers of distinct chemical composition might be used to control the accommodation of gases and their transport through the gas–liquid interface or alter the depth at which homogeneous catalyst molecules are held.

It is now well established that the bulk structures of many ILs exhibit microheterogeneity, with nanoseparation into polar and nonpolar domains.^30−33^ This behavior is promoted by pendant alkyl chains on either the cation or anion, which occupy the nonpolar domains, interpenetrated by polar domains consisting mainly of cation headgroups with attendant anions. The incorporation of alkyl chains is a very common motif in ILs used in practical applications, with variations in the chain length being used to fine-tune other desired physical properties, for example, liquid crystallinity.^34^

Microheterogeneity extends also to IL surfaces, with a clear tendency for longer alkyl chains to occupy the gas (or vacuum)–liquid interface preferentially in a range of common IL systems; this has been established through an array of physical techniques including, for example, angle-resolved X-ray photoelectron spectroscopy (ARXPS), low-energy ion scattering, Rutherford back scattering (RBS), metastable-atom electron spectroscopy, secondary-ion mass spectrometry (SIMS), neutron reflectivity, X-ray reflectivity, sum-frequency generation (SFG), and second-harmonic generation.^35−46^

Our own contribution to demonstrating this phenomenon has been through the development of the technique of reactive-atom scattering (RAS). In this method, reactive atomic projectiles are directed at a liquid surface and gas-phase products detected which are characteristic of the reaction with a specific functional group exposed at the liquid surface.^32,47−56^ In one variant, as will be used here, the products are detected by laser-induced fluorescence (hence RAS-LIF).^32,47,48,50,52−55^ An alternative, developed independently in the Minton group, is to use mass-spectrometric detection (RAS-MS).^49−51,54,56^ We have shown the power of the RAS-LIF and RAS-MS methods to analyze the extreme outer surfaces of a number of IL systems, including homologous series of imidazolium- and pyrrolidinium-based liquids, the coupled effects of variation of the anion, and even liquid-crystalline materials.^47−53^ The majority of this work has used ground-state oxygen, O(^3^P), atoms as the projectile, with the detection of OH (RAS-LIF) or inelastically scattered O, OH, and H_2_O (RAS-MS). Recently, we have also shown that RAS-MS can be extended to F(^2^P) projectiles, combined with the detection of HF, or DF for isotopically labeled samples, giving detailed site-specific information on the occupancy and orientation of cations at the surface.^56^

All the experimental methods above have strengths and weaknesses. They vary in their chemical specificity and penetration depth, i.e., what ultimately constitutes “the surface” of the liquid. Consequently, complementary molecular dynamics (MD) simulations have also played a very important role in this field.^30−32,45,57^ Unlike any single current experimental technique, they can, in principle, give a full molecular-level description of the interfacial region over the full range of depths. However, these simulations are ultimately only as reliable as the force fields and other aspects of the MD methodology used, so it is crucial to test their predictions against experimental observations. This is also an aspect on which we focus closely in this work.

The search for materials with tunable properties for a given application is beset by the rapidly diverging combinatorial possibilities arising from trial-and-error testing of all conceivable binary combinations of cations and anions. A promising, but as yet relatively underexplored, strategy is to generate mixtures of a pair (or other small number as a basis set) of ILs in different proportions.^58−61^ The progression in bulk properties and interesting variations in underlying nanostructure has been demonstrated, for example, in mixtures of short- and long-chain versions of a common cation combined with a common anion.^32^ We have also investigated the competition for surface sites between long and short chains in such mixtures based on the widely studied 1-alkyl-3-methylimidazolium cation (labeled [C_*n*_mim]^+^, where *n* is the length of the alkyl side chain), combined with the common anion bis(trifluoromethylsulfonyl)imide ([Tf_2_N]^−^). In a study that is technically closest to the current work, we showed that RAS-LIF can be used successfully to investigate the surfaces of [C_2_mim]_1–*x*_[C_12_mim]_*x*_ [Tf_2_N] mixtures.^32^ We found a clear nonstoichiometric preference for the longer chains to occupy the surface over most of the mole-fraction range. There were corresponding changes to the nanoscopic domain structure in the bulk, as determined experimentally by neutron and X-ray scattering, and complemented by extensive MD simulations. We have examined the applicability of fitting functions based on established physical models for the deviation from stoichiometric surface coverage and discussed how our RAS-LIF observations relate to other independent observations for sparser sets of related mixtures using ARXPS, RBS, and SIMS.^37,55,62,63^

An obvious alternative to combinations of different chain lengths is mixtures of distinct chemical functionality. A class of pure ILs generating interest in their own right is those containing fluorinated chains on either the cation or the anion.^64^ This is also the chemically novel aspect that we exploit in the current work. The well-known differences in molecular-level properties (volume, stiffness, polarity, and polarizability) induced by fluorination can be expected to lead to corresponding changes in the physical properties [density, viscosity, hydrophobicity, surface tension (ST), etc.] relative to their alkyl analogues. Both types of chain can be present in the same material; this can be achieved either in a single-component IL, for example, by attaching different chain types to the cation and anion, respectively, or by attaching both types of chain to the cation.^65^ Variations in properties can then be investigated by synthesizing different materials with different combinations of chain type and length. Alternatively, two distinct ILs, one containing alkyl chains and the other fluoroalkyl chains, can be mixed in different proportions.^66^ It is this latter approach that is of particular interest here.

Recent work has begun to unravel in more detail the bulk structure and properties of fluorinated ILs and their mixtures.^64−76^ The existence of fluorinated domains in various fluorinated IL systems has been predicted through MD modeling and confirmed experimentally through neutron and X-ray scattering. In particular, MD simulations of mixtures of alkyl- and fluoroalkylimidazolium ILs by Hollóczki et al. revealed *bulk* “triphilic” behavior.^66,77^ They identified three distinct domains in mixtures with essentially equal (C8) alkyl and fluoroalkyl cationic chain lengths combined with a common anion (bromide); that is, polar (cation headgroups and anions), nonpolar alkyl, and nonpolar fluorous domains. They speculated that these unusual characteristics might allow ”smart” liquids to be developed whose properties could be switched in predictable ways through changes in composition or other stimuli such as temperature.

The focus of the current work is the even more sparsely investigated field of the *surfaces* of mixed alkyl/fluoroalkyl IL systems. Information on the specific competition for surface sites between alkyl and fluoroalkyl chains has been derived from ST measurements in ILs consisting of alkylimidazolium cations and perfluorobutyl sulfonate anions, and correlated with MD simulations of the corresponding structural changes.^78^ The perfluorinated butyl chains of the anions appear to dominate the surface for cationic alkyl chains shorter than C4, with the increase in penetration by longer alkyl chains. The ST is minimized for C8, when the combined overall surface density of alkyl and fluoroalkyl chains is maximized. It increases again for longer alkyl chains, which become dominant at the surface. Most recently, Heller *et al.* have investigated the surfaces of mixtures of varying mole fractions of butyl and 3,3,4,4,4-pentafluorobutylimidazolium cations combined with a common [PF_6_]^−^ anion using ARXPS and through ST and surface light-scattering measurements.^79,80^ The topmost layers were found to be enriched in pentafluorobutyl chains, with the deviation from stoichiometry largest for the most dilute mixture examined (10% pentafluorobutylimidazolium). The enrichment was enhanced at lower temperatures, while the system remained liquid. The surface preference for fluorinated chains has been shown to extend to competition with methoxy-functionalized ILs.^81^

As a relatively minor part of our previous work,^55^ intended to demonstrate the universality of the functional form of the deviations from stoichiometry in different types of IL mixtures, we presented preliminary, proof-of-principle RAS-LIF data for a single fluoro/alkyl IL mixture. We expand the examination of the surface properties of this class of material considerably in scope and explore it in much more depth here. Thus, [C_8_mim][Tf_2_N] was mixed with its semiperfluorinated analogue (1*H*,1*H*,2*H*,2*H*-perfluorooctyl)-3-methylimidazolium bis(trifluoromethylsulfonyl) amide (which we label [C_8_mimF_13_][Tf_2_N]). The measured *deficit* of OH and hence of exposed alkyl chains relative to the expectations from stoichiometry implied, indirectly, that there must be a corresponding *excess* of fluoroalkyl chains at the surface. We present here a more extended, systematic investigation of mixed alkyl/fluoroalkyl IL systems based on the same fluorinated cation ([C_8_mimF_13_]^+^) mixed with alkylated cations of varying chain lengths ([C_*n*_mim]^+^ with *n* = 4–12). We address the question of whether the apparent higher intrinsic surface preference for fluorinated over alkyl chains of the same length might be counterbalanced by the also known increase in surface activity of alkyl chains with the increase in chain length.^50^ We examine the extent to which the RAS-LIF observations are reproduced by large-scale MD simulations and what additional insights they provide into the molecular-level organization of the surfaces of these interesting materials.

## Methods

### Materials

**Figure 1:**
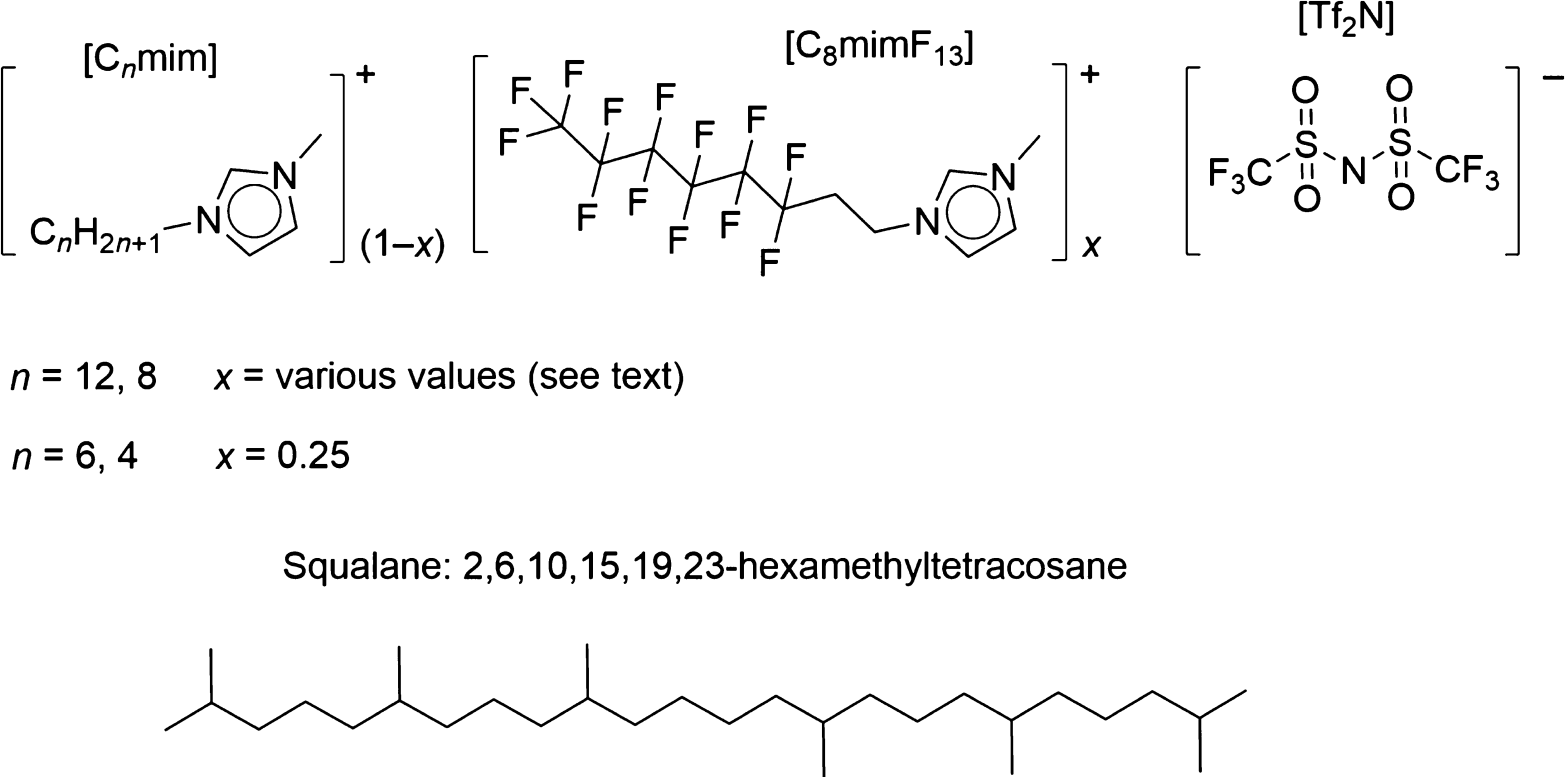
Molecular structures of the components of the fluoroalkyl ionic–liquid mixtures, along with the reference liquid, squalane. *x* indicates bulk mole fraction of the partially fluorinated [C_8_mimF_13_][Tf_2_N] component.

The molecular structures of the liquids used in this study are shown in Figure 1. Note the inclusion, for practical synthetic reasons, of a −CH_2_CH_2_– linker next to the ring in [C_8_mimF_13_][Tf_2_N]. [C_8_mimF_13_][Tf_2_N] was mixed with [C_*n*_mim][Tf_2_N], where *n* = 4, 6, 8, or 12—these mixtures are written here as [C_*n*_mim]_(1–*x*)_[C_8_mimF_13_]_*x*_[Tf_2_N], where *x* is the bulk mole fraction of the [C_8_mimF_13_][Tf_2_N] component. For *n* = 8 and 12, a full range of *x* was studied, whereas for *n* = 4 or 6, a fixed mole fraction of *x* = 0.25 was used.

The syntheses of [C_8_mim][NTf_2_] and [C_8_mimF_13_][NTf_2_] have been described previously,^55^ as have those for [C_4_mim][Tf_2_N] and [C_12_mim][Tf_2_N].^32,56^ [C_6_mim][NTf_2_] was prepared in a directly analogous manner from freshly distilled 1-methylimidazole and 1-bromohexane. Commercial samples of [C_8_mim][Tf_2_N] (99%) and [C_12_mim][Tf_2_N] (99.9%), used for confirmatory RAS-LIF measurements at *x* = 0, were supplied by IoLiTec. Water (prior to additional degassing, below) and halide content for these liquids are given in Supporting Information 1.1. The branched hydrocarbon squalane was purchased from Sigma-Aldrich (99%) and was used as a reference liquid in the RAS-LIF experiments. All liquids were degassed overnight under vacuum (<10^–6^ mbar) to remove volatile components, prior to measurements being taken.

### Surface Tension

The ST of the [C_8_mim]_(1–*x*)_[C_8_mimF_13_]_*x*_[Tf_2_N] mixtures were recorded using a Krüss DSA100 tensiometer using the pendant drop method. The instrument has a resolution of 0.01 mN m^–1^ and an accuracy of 0.3 mN m^–1^. For all compositions, eight or more measurements were recorded and averaged.

### RAS-LIF

**Figure 2:**
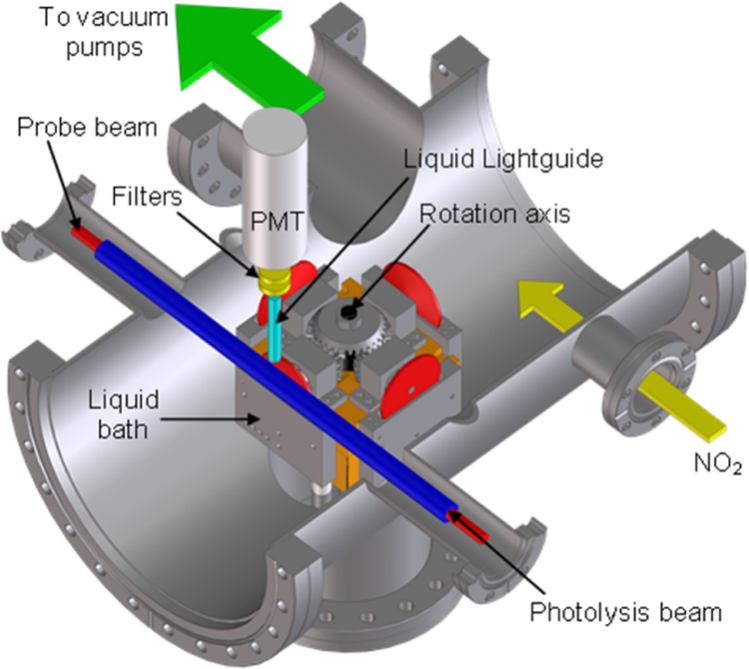
Schematic diagram of the RAS-LIF apparatus. The central, four-wheel carousel could be rotated about its central axis, which allowed each of the liquid wheels to be presented to the laser beams for study.

The RAS-LIF apparatus is shown in outline in Figure 2. A brief overview is given here as detailed descriptions have been presented previously.^47,48,50,52−55^ The liquid surface was created by rotating a partially immersed wheel (diameter 5 cm, speed 30 rpm) in a bath of liquid. This dragged a liquid film onto the wheel and created a continually refreshed liquid surface. In the previous work, we have shown that the rotation speed has no discernible influence on the results, even for liquid–crystalline IL materials with much a longer-range order than those used here.^52^ This is consistent with estimated distances for one-dimensional diffusion of ions away from the surface in a suitable fraction of the rotation period, which, using typical diffusion coefficients for representative ILs,^82^ are around three orders of magnitude larger than the effective thickness of the surface region as determined by MD simulations (see below). RAS-LIF results using the same approach for related IL mixtures containing long and short alkyl chains are also corroborated by other surface-sensitive measurements on static samples.^32,55^ We are therefore confident that the surface formed represents an equilibrium structure. A carousel, which housed four such wheels, allowed accurate relative measurements to be made by being able to switch between wheels (by rotating the carousel 90° successively about its central axis) without breaking the vacuum. Passing parallel to one of the wheels (distance to the center of the beam 6.7 mm), a pulsed photolysis laser beam (355 nm, 80 mJ, ∼6 ns fwhm) dissociated a low pressure (1 mTorr) of NO_2_ precursor gas above the liquid surface. O(^3^P) atoms were generated with a distribution of translational energies (mean 16 kJ mol^–1^, fwhm 26 kJ mol^–1^).^83^

Some of the O-atoms incident at the liquid surface reacted to form OH via abstraction. For energetic reasons, as discussed further below, the OH products are expected to be derived almost exclusively from secondary aliphatic C–H bonds, and therefore, the amount of OH detected is correlated directly with the surface exposure of −CH_2_– groups in the alkyl chains. OH in the gas phase was detected via LIF excited on the A–X (1,0) band by a pulsed probe laser beam (∼283 nm, 250 μJ, ∼5 ns fwhm) that counter-propagated the photolysis beam. The OH fluorescence was collected by a liquid light guide and, after optical filtering to isolate the A–X(1,1) band, directed onto a photomultiplier tube to provide a signal which is proportional to the number density of OH molecules in the probe volume. For most measurements of relative number densities, OH was probed on the most intense, Q_1_(1), line. A small background contribution to the OH density resulting from direct photolysis of a contaminant in the NO_2_ precursor, thought to be HONO, was subtracted for all liquids studied, as described previously.^50,84^

During RAS-LIF measurements, all liquids were temperature-controlled to 47 ± 2 °C, chosen to lower the viscosity of the fluorinated ILs. This resulted in a better-quality, uniform liquid film on the surface of each of the carousel wheels.

### Molecular Dynamics

MD simulations of the ILs were performed in GROMACS 5.1.2 using the CL&P extensions^57,85−90^ to the OPLS-AA force field.^91−98^ Additional parameters describing the intramolecular interactions around the CH_2_–CH_2_ linker unit in [C_8_mimF_13_]^+^ were used,^85,90^ along with the perfluoroalkyl parameters developed by Watkins and Jorgenson.^98^ Simulations were performed for multiple compositions in the C8 system and at *x* = 0 and *x* = 0.25 for the C4, C6, and C12 systems. The majority of the simulations used 800 ion pairs; an additional two liquids, pure [C_8_mimF_13_][Tf_2_N], and a [C_8_mim]_0.5_[C_8_mimF_13_] _0.5_[Tf_2_N] mixture were simulated using 1600 ion pairs to establish if the simulated interface was affected by the finite system size. A detailed description of the procedure for each simulation is given in Supporting Information 2.1 and is summarized briefly here.

The first step was the simulation of the bulk liquid. Ions, in a single conformation of each type, were randomly packed into a cubic box with length 7–9 nm. Following a steepest-descent energy minimization, each liquid was simulated under *NPT* conditions for typically ∼0.5 ns using a Berendsen barostat (1 bar) and velocity-rescaling thermostat at 500 K. Subsequently, the system was typically propagated for 4 ns using a Parrinello–Rahman barostat (1 bar) and a velocity-rescaling thermostat at 320 K, the temperature of the RAS-LIF experiments in this work. The simulated densities are given in Supporting Information 2.1; they systematically overestimated the measured bulk density of the liquids by <6%, which is not unexpected for the force field used.^90^

The final frame from the bulk runs was extended in the *z*-dimension by a factor of three. This allowed for the simulation of a slab, typically 8–10 nm thick, with two vacuum interfaces, while still using 3D periodic boundary conditions. Under *NVT* conditions, repeated cycles of 5 ns at 320 K, followed by 5 ns at 500 K were used to equilibrate the slab out to ∼80 ns, when a final 10 ns at 320 K was run. Typically, MD trajectories were therefore run for 90 ns overall, with results generally being reported here, for reasons described below, from averages over the final ∼45 ns excluding the 500 K annealing cycles.

A solvent-accessible surface-area (ASA) algorithm,^99^ which is implemented as part of GROMACS, was used to determine the areas of each atom type exposed at the liquid surface (at both interfaces). Extensive characterizations were carried out for different unique atom types, including different positions along the side chains. Of particular interest were the secondary H atoms because they are known to be the principal source of OH in the RAS-LIF experiments, as discussed further below.^50^ Thorough exploratory analyses confirmed that a probe particle radius of 0.15 nm, close to the accepted van der Waals radius for O atoms,^100^ was sufficient to prevent any significant unwanted contributions from voids within the bulk liquid. Counting of atoms whose exposed area exceeded a selected threshold level was also explored as explained in Supporting Information 2.2. It was used in some preliminary tests of equilibration and convergence, but was found to be prone to artifacts for quantitative comparison of different atom types. The principal results reported here are therefore the summed exposed areas of atoms of a given type, normalized as appropriate to the total exposed surface area for a given liquid as explained below.

The ASA analysis was carried out for selected frames in the relevant MD trajectory. To account for the (artificially, on account of the classical force fields)^101^ slow MD dynamics of the IL surface relative to the simulation time step, a block analysis^102^ (see Supporting Information 2.3) was used to determine the effective relaxation time of the surface. For several of the simulated liquids, the average surface relaxation time of the secondary H-atom count was found to be ∼0.4 ns. Therefore, the trajectories were sampled every 0.4 ns to produce broadly uncorrelated frames, with the average and standard error calculated in the conventional way. This resulted in a minimum of 17 ns of simulation time (42 frames) for each liquid from which the average surface-accessible area of selected atom types was calculated.

System size effects were investigated by comparing the 1600 and 800 ion-pair systems for [C_8_mim]_*x*_[C_8_mimF_13_]_(1–*x*)_[NTf_2_] with *x* = 0 and *x* = 0.5. The general structure of the interfaces was very similar, other than some weak density oscillations which reached the middle of the slab for the 800 ion-pair systems but not for those with 1600 ion pairs (see Supporting Information 2.4). The results for the areal density of surface hydrogen atoms for different system sizes also agreed within their respective errors (see Supporting Information 2.5).

The surface-hydrogen density for each liquid was monitored as the simulation progressed; this was the basis for selecting only the final ∼45 ns of each trajectory for further analysis because by this time, results for a wide range of liquids no longer varied within a small tolerance (see Supporting Information 2.5).

## Results

### Surface Tension

**Figure 3:**
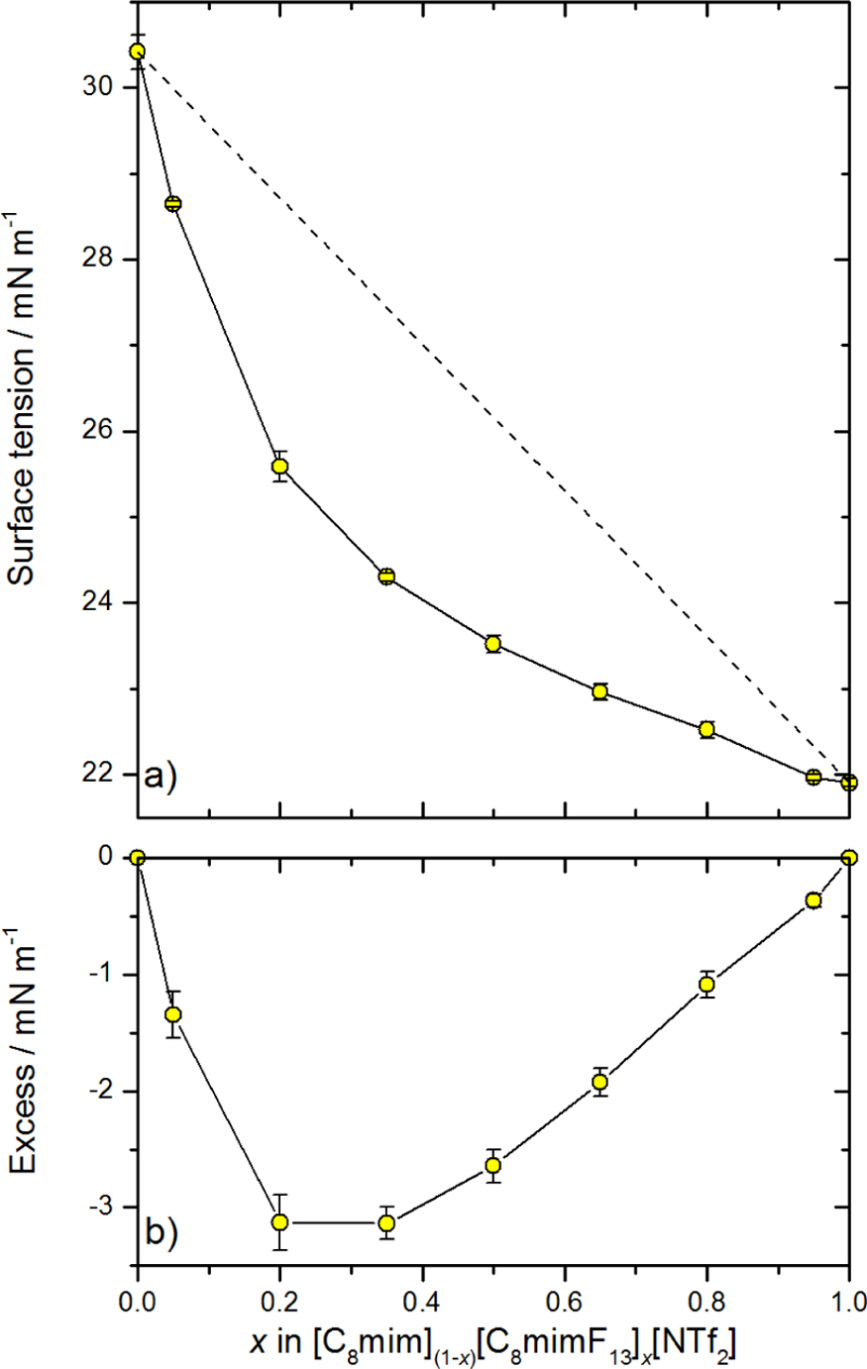
(a) ST data for [C_8_mim]_(1–*x*)_[C_8_mimF_13_]_*x*_[Tf_2_N] mixtures. The dashed line illustrates a linear-mixing model (eq 1). (b) Excess ST, the difference between ideal and observed behavior, which is negative here. All errors are 95% CL; derived from the repeatability of the measurements and not the accuracy of the instrument, which is a much smaller source of error.

The STs of the pure components of the [C_8_mim]_(1–*x*)_[C_8_mimF_13_]_*x*_[Tf_2_N] system have been reported previously;^55,103^ for the mixtures, the new measurements here are shown in Figure 3a (values are tabulated in Supporting Information 3.1.). As *x* increases, it is clear that the ST decreases to a greater extent than would be expected from a simple linear-mixing model based on the bulk mole fractions of the two ILs (eq 1)
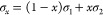
1where σ_1_ and σ_2_ are the STs of the pure components (1 refers to alkyl and 2 to fluoroalkyl) and σ_*x*_ is the measured ST of the mixture. This relationship is indicated by the dashed line in Figure 3a.

Figure 3b illustrates the deviation from the linear-mixing model by plotting the excess ST (difference between the ST of the mixture and that predicted from linear mixing) for each composition. The largest deviation in the surface excess, which is negative here, occurs in the composition range between *x* = 0.2 and 0.4.

For the purposes of comparison with the RAS-LIF data and MD simulation to follow, we can invert the logic of eq 1 to assert that the observed ST is a linear combination of the STs of the pure liquids weighted by the *surface* mole fractions of the fluoro (denoted *x*_s_) and alkyl (1 – *x*_s_) components, respectively, that is
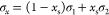
2a

Note that this is an implicit equation for the unknown surface mole fractions in terms of the measured ST at a known bulk composition, *x*, and is equivalent to
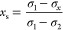
2b

A similar model (other than being expressed in terms of volume rather than mole fractions, which will be similar here) gives a reasonable fit to measured ST data for mixtures of long- and short-chain alkyl ILs.^55^ As discussed in this previous work, the best-fit value of an adjustable weighting parameter allowing for higher-order cross terms was close to zero.

**Figure 4:**
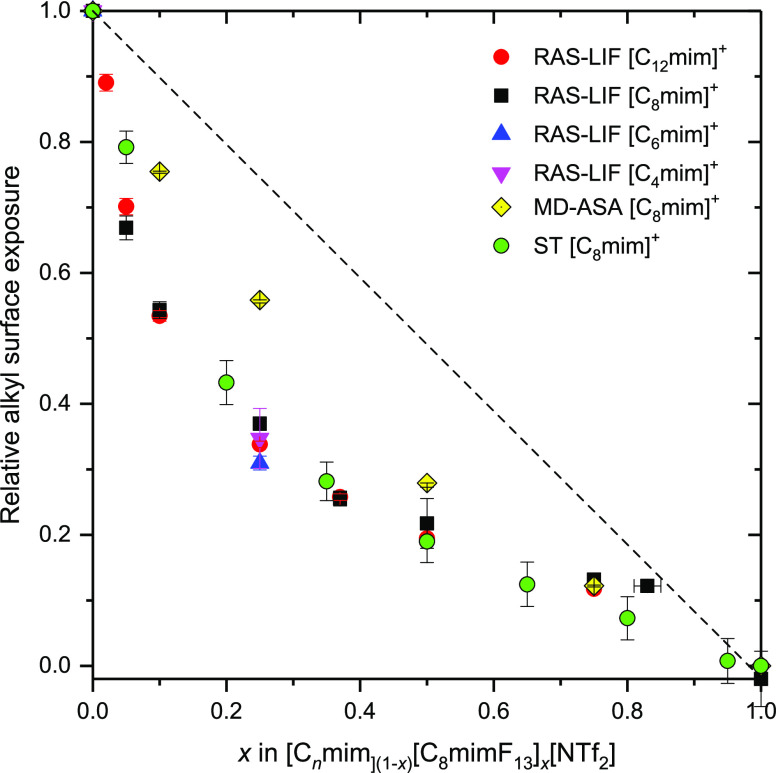
Comparable relative measures of surface exposure of the alkyl component in [C_*n*_mim]_(1–*x*)_[C_8_mimF_13_]_*x*_[Tf_2_N] mixtures. ST = alkyl surface mole fraction (1 – *x*_s_) as defined through eqs 2a and 2b for C8 mixtures only (green circles). RAS-LIF = normalized reactivity to produce OH as defined in eq 3 (red circles C12 mixtures; black squares C8 mixtures; blue triangles C6 mixtures; and magenta triangles C4 mixtures). MD-ASA = relative fraction of the surface area consisting of reactive secondary-hydrogen atoms for C8 mixtures only (yellow diamonds). The dashed line is the prediction for linear mixing in all cases. All experimental error bars 95% CL. A representative *x*-error bar, reflecting the precision of preparing the mixtures, is shown for *x* = 0.82. The confidence limits for the MD ASA analyses correspond to different assumptions about the alkyl chain positions which contribute, as described in the text, which makes only marginal differences here.

The resulting values of (1 – *x*_s_) (i.e., the implied fraction of the alkyl component at the surface) from eqs 2a and 2b are included as a function of the bulk composition, *x*, in Figure 4. They show the expected strong negative deviation from the linearity that would have been predicted by eq 1 (dashed line) (the other data with which they are compared are introduced below.)

### RAS-LIF

**Figure 5:**
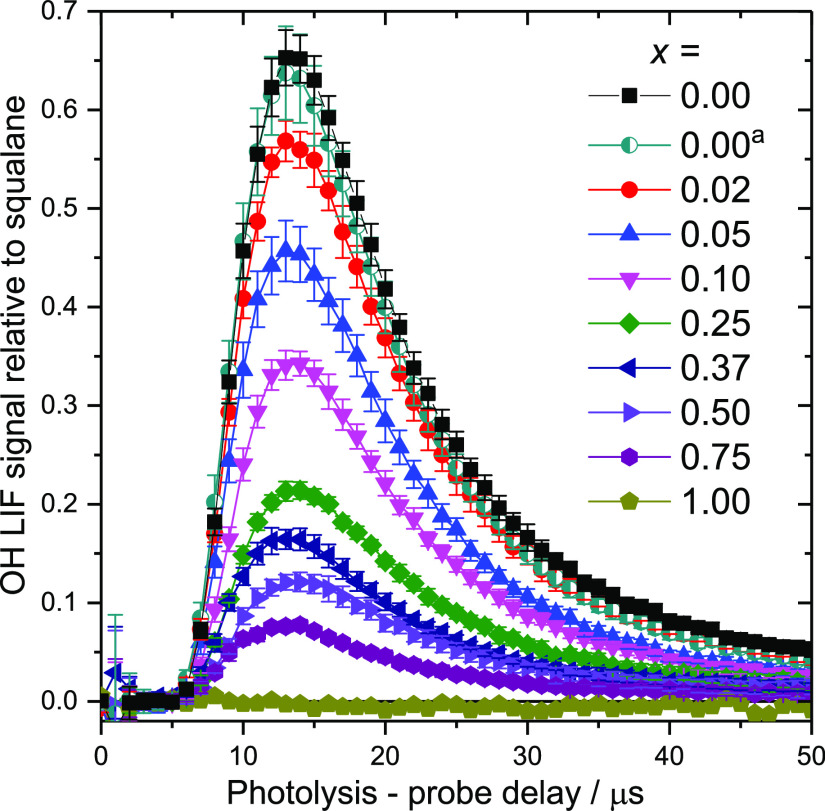
OH appearance profiles (OH density as a function of photolysis-probe delay) from [C_12_mim]_(1–*x*)_[C_8_mimF_13_]_*x*_[Tf_2_N] for varying *x*, as shown. Signals are normalized to those from the squalane reference liquid. ^a^Measurement of commercial sample, confirming reproducibility of data. Error bars are 95% CL.

Figure 5 shows, as a representative example, the detected OH density from the [C_12_mim]_(1–*x*)_[C_8_mimF_13_]_*x*_[Tf_2_N] system of mixtures as a function of delay between the photolysis and probe laser pulses. Similar data for [C_8_mim]_(1–*x*)_[C_8_mimF_13_]_*x*_[Tf_2_N] mixtures have been reported previously^55^ and those for the C4 and C6 mixtures are given in Supporting Information 4.1. These OH appearance profiles are normalized to the peak of the corresponding profile from squalane. The squalane reference data were recorded immediately after those from each mixture of interest. Typically, 10 such individual relative measurements were made for each mixture and averaged. This approach reduced the effect of unavoidable minor variations in the experimental conditions that affect the magnitude of the LIF signal, providing a precise, consistent measurement relative to the reference liquid. An additional profile was measured for a commercial sample of pure [C_12_mim][Tf_2_N] (i.e., *x* = 0). This gave essentially identical results to the sample synthesized in-house, used to make the mixtures for other values of *x*.

The OH rotational distribution was also determined for *x* = 0 and 0.5 by taking the LIF excitation spectrum (LIF intensity as a function of probe laser wavelength) with the photolysis-probe delay fixed at the peak of the OH appearance profile. It was found not to change significantly with *x* (see Supporting Information 5.1 and 5.2). This simplifies the interpretation of Figure 5 in that variations in the OH signal are related directly to an increase in total OH density, and by inference, the surface alkyl chain exposure of the methylene units and not due to a redistribution of OH rotational populations. This assumption has been verified previously.^50^ The same assumption was made for the C4 and C6 chain length mixtures, where rotational distributions were not recorded due to the lower signal levels.

The appearance profiles in Figure 5 all have essentially the same peak arrival time (other than for *x* = 1.00, for which there is effectively no real signal—see below), implying that the most-probable scattered OH velocity is the same for all liquid mixtures. Therefore, a density-to-flux transformation was not required and the OH densities in Figure 5 can straightforwardly be integrated between fixed limits to yield a single value representative of the OH yield from each mixture (see Supporting Information 6.1–6.4); we term these integrated values the *relative reactivity* with respect to squalane.

**Figure 6:**
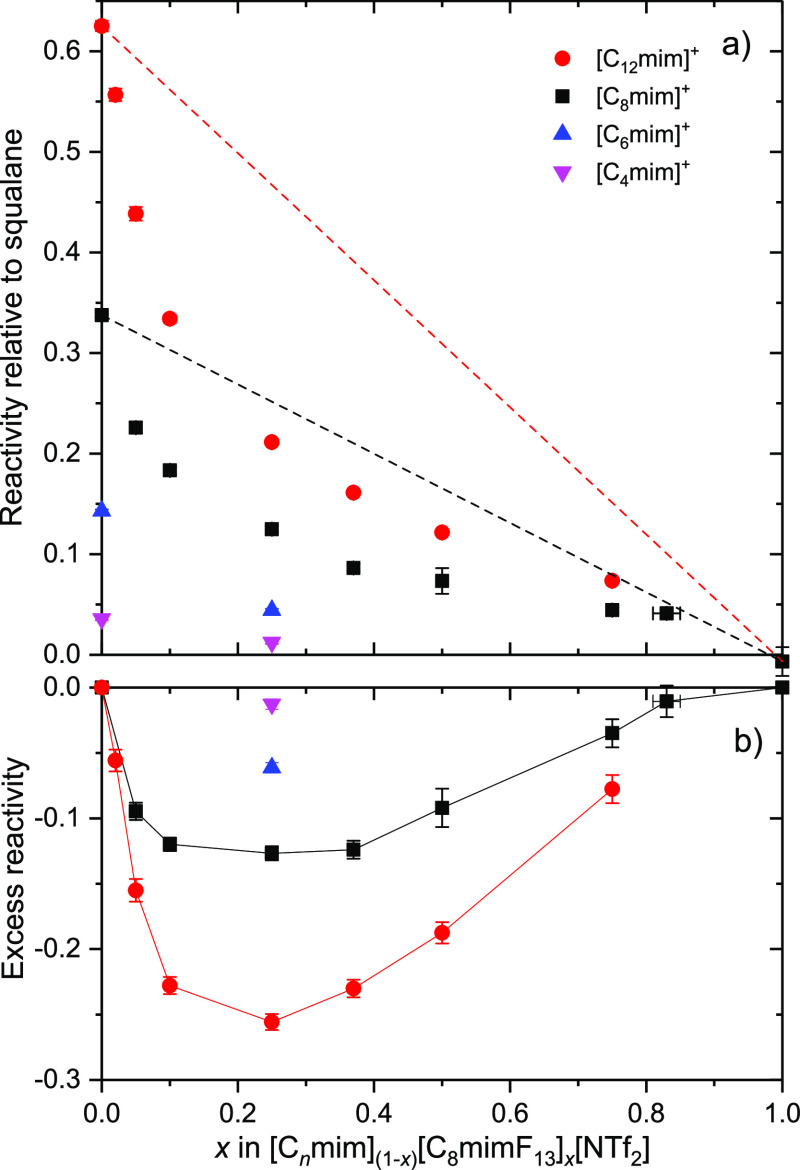
(a) RAS-LIF reactivity relative to squalane for the neat liquids and for [C_*n*_mim]_(1–*x*)_[C_8_mimF_13_]_*x*_[Tf_2_N] mixtures, with chain lengths *n* as shown. The dashed lines show the behavior expected from a linear mixing law for C8 (black dashed line) and C12 (red dashed line). (b) Excess reactivity, which is the difference (not renormalized) between the reactivity of a mixture and the linear mixing prediction. All errors bars are 95% CL. A representative *x*-error bar, reflecting the precision of preparing the mixtures, is shown for *x* = 0.82.

Figure 6 summarizes the RAS-LIF relative reactivities, normalized to squalane, for all chain lengths and compositions studied. Some observations and trends are immediately clear.

First, for *x* = 1, the reactivity is effectively zero; that is, no measurable OH is detected. This implies that, in addition to the well-established absence of reactivity of the imidazolium-ring H atoms,^32,47,48,50,53^ the −CH_2_CH_2_– linker in [C_8_mimF_13_][Tf_2_N] is not amenable to H-abstraction by O(^3^P). We return to the relative contributions of different C–H bond types below.

Second, for all mixtures, as *x* increases there is a decrease in reactivity larger than that expected from stoichiometry for linear mixing, which is indicated by the dashed lines for C12 (red) and C8 (black) in Figure 6a. This implies that [C_*n*_mim]^+^ ions are statistically underrepresented relative to their bulk composition at the surface for all *n*. The corresponding (negative) excesses in relative reactivity are shown in Figure 6b.

Third, by comparing the reactivities of the C8 and C12 mixtures, there is a larger *absolute* deviation from linear mixing for C12 than for C8; that is, as the alkyl chain length increases, a larger number of reactive sites are displaced from the surface by a given mole fraction of semiperfluorinated chains. However, the absolute OH yield for the pure C12 liquid is already known to be larger than that for the pure C8 (as confirmed by the behavior at *x* = 0 in Figure 6a).^50^ This can be taken into account by normalizing the reactivity of each mixture to the reactivity of the corresponding pure liquid at *x* = 0 via eq 3
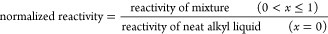
3

Note that this produces a relative measure of the fraction of the alkyl component at the surface formally equivalent to eq 2b, but directly in terms of the measured OH yield from the alkyl component for which no further assumption of linearity is required (because the OH yield from the fluoro component is effectively zero).

The normalized reactivities are included in Figure 4 as a function of *x* for different chain lengths. Interestingly, the reactivities for C8 and C12 are nearly identical across the whole range in *x* when expressed in this relative form. The more restricted RAS-LIF measurements for C6 and C4 at a mixing ratio of *x* = 0.25 also suggest essentially the same relative reduction in reactivity as a proportion of that in the pure liquid on introduction of [C_8_mimF_13_]^+^ ions. Moreover, the agreement with the surface composition based on the ST data as derived via eqs 2a and 2b for C8 mixtures is rather good. We return to this in the Discussion below.

**Figure 7:**
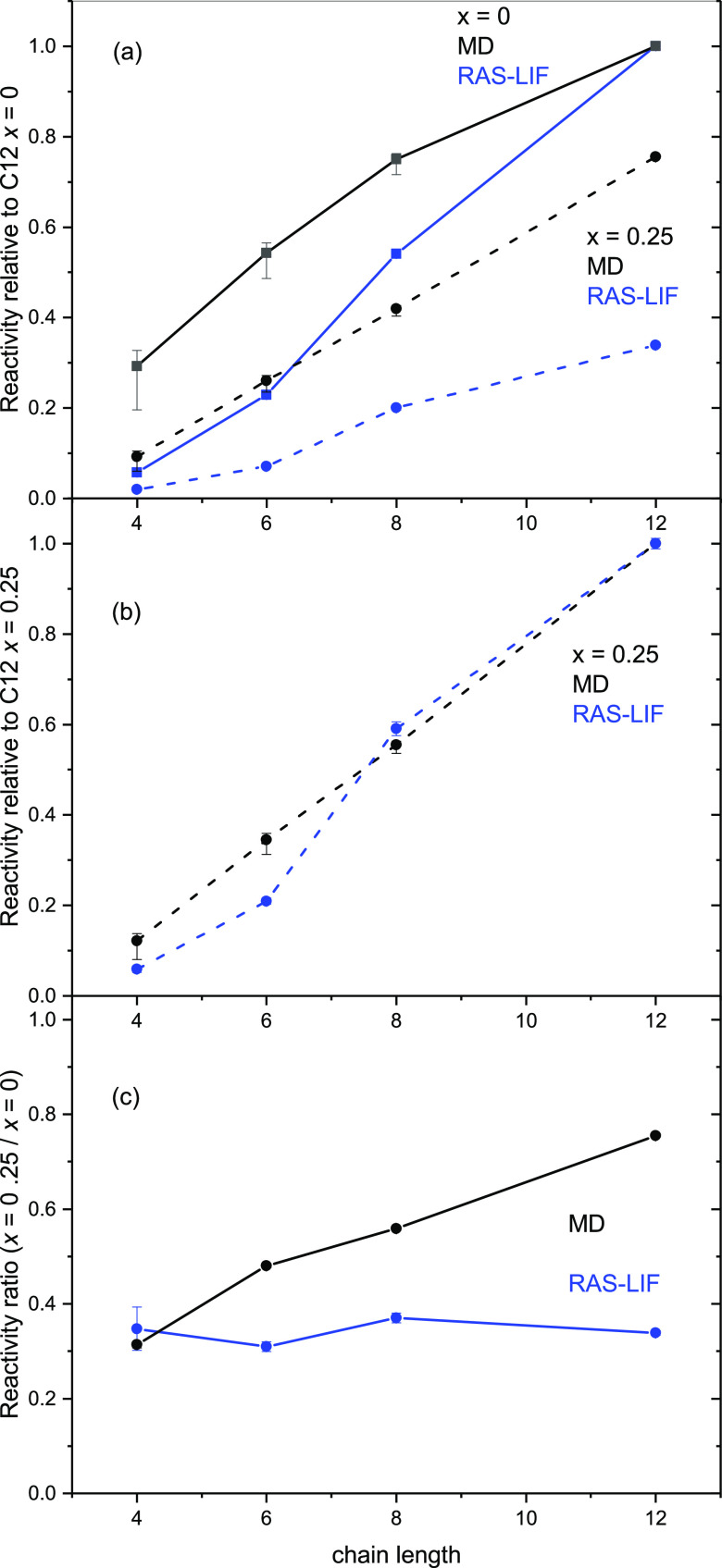
(a) Relative reactivities from either RAS-LIF (OH signals, blue) or MD ASA (proportion of the exposed surface covered by secondary hydrogen, black) for pure alkyl liquids (*x* = 0, squares, solid lines) or [C_8_mim]_0.75_[C_8_mimF_13_]_0.25_[Tf_2_N] mixtures (*x* = 0.25, circles, dashed lines), as a function of alkyl chain length, *n*. Both RAS-LIF and MD ASA results have been normalized to those for pure C12 (i.e., *x* = 0, *n* = 12). (b) RAS-LIF and MD ASA results [symbols and lines as in (a)] for the same *x* = 0.25 mixtures renormalized at *x* = 0.25, *n* = 12. (c) Ratio of reactivity for *x* = 0.25 to *x* = 0 for RAS-LIF (blue) and ASA-MD (black). All experimental errors are 95% CL. The confidence limits for the ASA-MD analyses correspond to different assumptions about the alkyl chain positions which contribute, as described in the text. Note that these largely cancel in (c) because the effects of this assumption on *x* = 0.25 and *x* = 0 are correlated.

The variations with *n* for the pure liquids (*x* = 0) and the *x* = 0.25 mixtures are presented in an alternative form in Figure 7. For the purposes of further comparison with the MD simulations (see below), the RAS-LIF reactivities have been renormalized in Figure 7a to the result for pure [C_12_mim][Tf_2_N] (i.e., *x* = 0 for *n* = 12). All the other RAS-LIF measurements retain their correct relative values directly from the experiment. For the pure alkyl liquids, the strong increase in alkyl chain exposure with the increase in *n*, already well known from previous work,^48,50^ is reproduced here. Because the point at which any of the MD simulations is normalized to the RAS-LIF measurements is arbitrary, we show a second choice in Figure 7b in which this is done for the *x* = 0.25 mixture for *n* = 12, to which we will return below.

The significant absolute reductions in alkyl-chain exposure between *x* = 0 and *x* = 0.25 for all *n* are apparent in Figure 7a. The *proportional* decreases between *x* = 0 and *x* = 0.25 are independent of any choice of normalization in either the measurements or the simulations. They are plotted in Figure 7c, where it is obvious that the RAS-LIF reactivities for the *x* = 0.25 mixtures are essentially a constant proportion, of around ∼35%, of those for the pure alkyl liquids.

### MD Simulations

**Figure 8:**
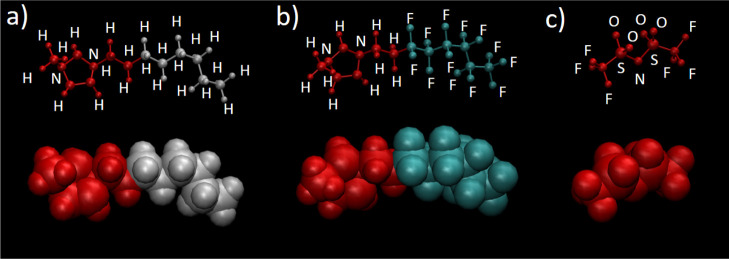
Color scheme used for the MD snapshots in Figures 9–12. (a) [C_*n*_mim]^+^ (in this case *n* = 8), (b) [C_8_mimF_13_]^+^, and (c) [Tf_2_N]^−^. For each species, the lower structure is a space-filling representation (as used in later figures) defined by van der Waals radii. The fluoroalkyl chains on the cation (C3–C8) are colored cyan, the equivalent chains on the alkyl cation are colored gray, and all other atoms are colored red. To ease rapid identification, the corresponding ball-and-stick representations are shown above in the same orientation and on the same scale. The same color scheme is used, with atom types (other than C, implied for unlabeled atoms) labeled explicitly.

We present first some selected snapshots at fixed points along MD trajectories to give a qualitative visual sense of the results of the MD simulations. These are generally the final frames of a given simulation run but are representative of typical, fully equilibrated samples, as established in the Methods section above and demonstrated in detail in Supporting Information 2.4 and 2.5). A common color coding is used throughout, selected to highlight the distinction between the charged regions made up of cationic headgroups and [Tf_2_N]^−^ anions (both shown in red) and the two potentially distinct nonpolar regions consisting of regular alkyl chains (gray) or fluoroalkyl chains (cyan), as also illustrated in Figure 8. For reasons introduced above and developed below, the first two members of all the alkyl chains and the −CH_2_CH_2_– linker in [C_8_mimF_13_]^+^ are color-coded as part of their respective headgroups.

**Figure 9:**
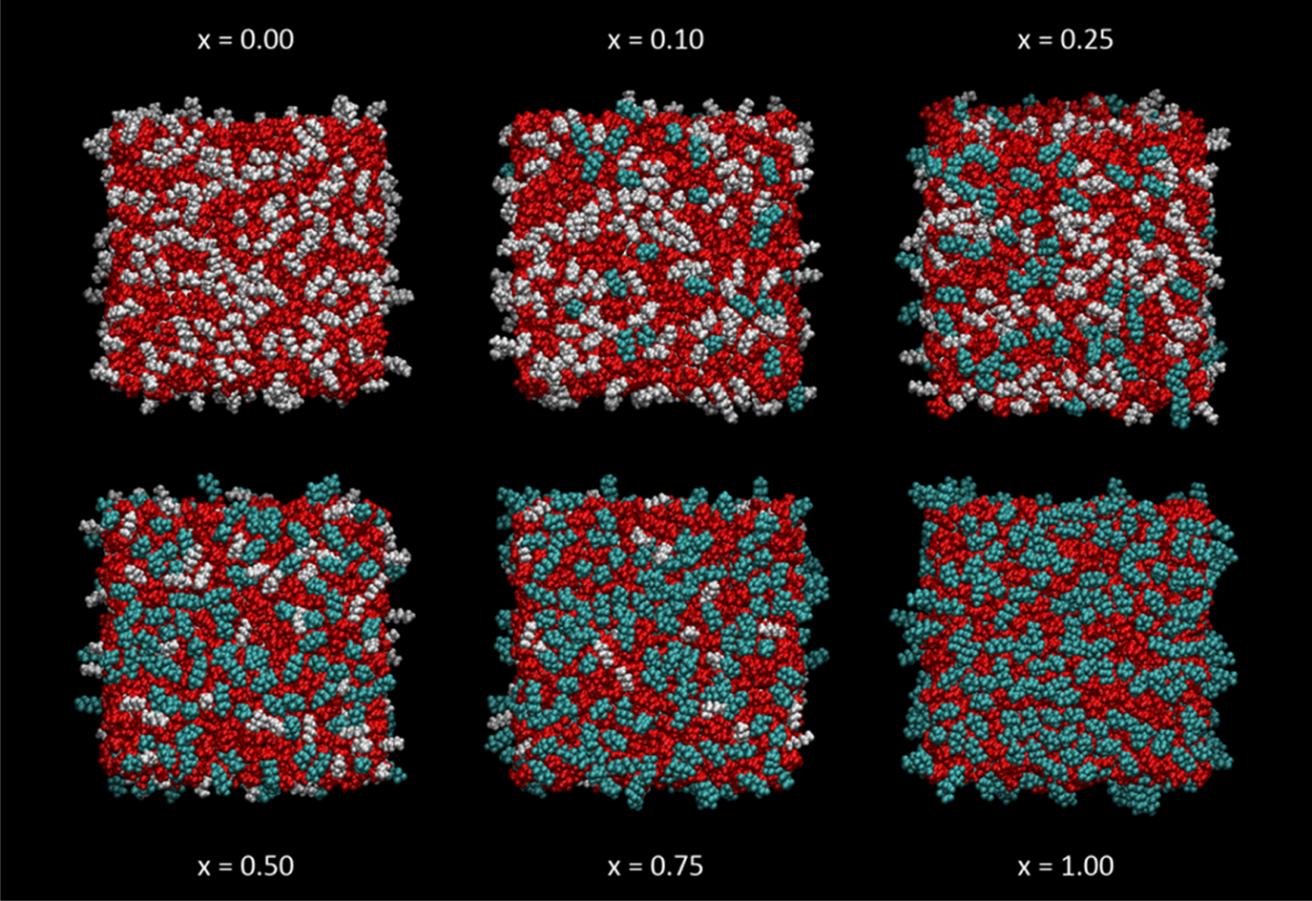
Top-down view of representative single MD snapshots of [C_8_mim]_(1–*x*)_ [C_8_mimF_13_]_*x*_ [Tf_2_N] mixtures; *x* as indicated. Color scheme as in Figure 8.

**Figure 10:**
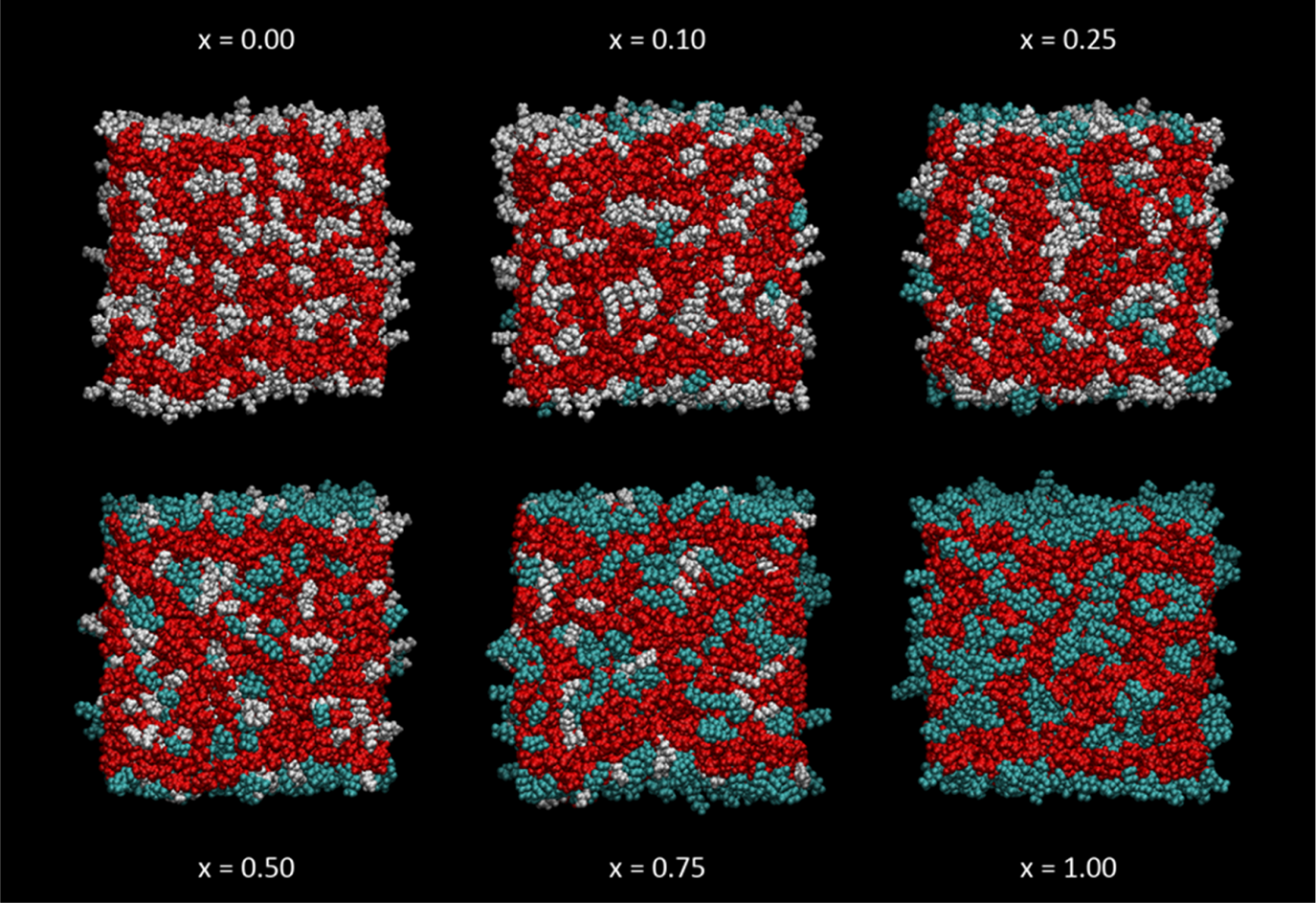
Side view of representative single MD snapshots of [C_8_mim]_(1–*x*)_ [C_8_mimF_13_]_*x*_ [Tf_2_N] mixtures; *x* as indicated. Color scheme as in Figure 8.

The variation in appearance with mole fraction, *x*, in [C_8_mim]_(1–*x*)_[C_8_mimF_13_]_*x*_[Tf_2_N] mixtures is shown in Figures 9 (top-down view) and 10 (side view). For this alkyl chain length, the surface is heavily populated by nonpolar cation chains throughout the full range of *x*, with a clear sublayer (Figure 10) composed almost entirely of polar headgroups and anions. Fluoroalkyl chains progressively displace alkyl chains from the surface layer as *x* increases. It is obvious even by qualitative inspection that the fluoroalkyl chains have a higher surface preference than the alkyl chains, with, for example, considerably more than half of the exposed chains being fluoroalkyl in the *x* = 0.5 frame.

**Figure 11:**
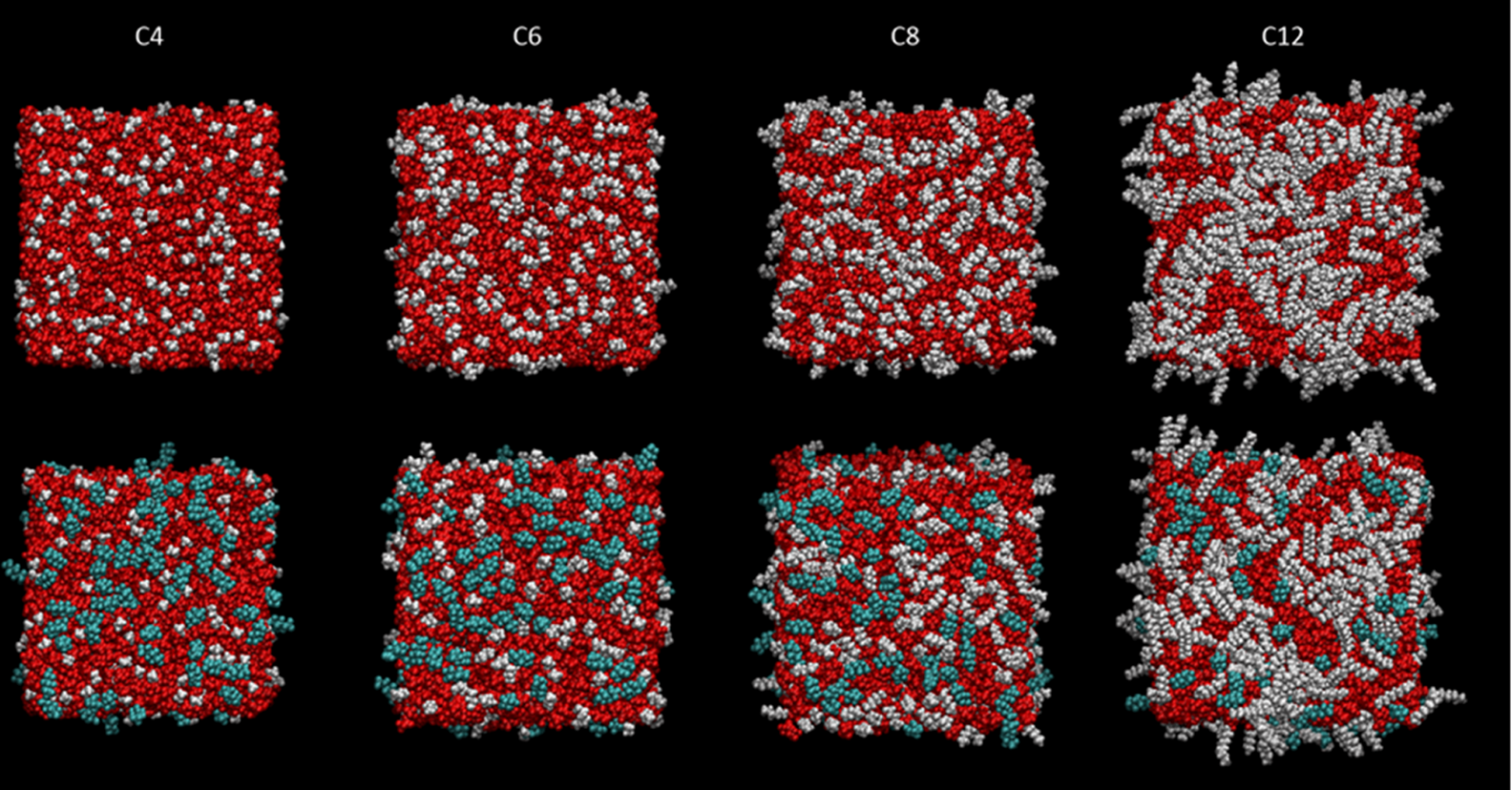
Top-down view of representative single MD snapshots of pure [C_*n*_mim][Tf_2_N] (upper row) and [C_8_mim]_0.75_ [C_8_mimF_13_]_0.25_ [Tf_2_N] mixtures (lower row) for *n* = 4, 6, 8, and 12. Color scheme as in Figure 8.

The corresponding trends with chain length in the [C_*n*_mim] cation are shown in Figure 11 (top-down view) and 12 (side view). In each case, the pure [C_*n*_mim][Tf_2_N] material (i.e., *x* = 0, upper rows) is compared with the [C_*n*_mim]_0.75_[C_8_mimF_13_]_0.25_[Tf_2_N] mixture (lower rows). As expected from previous work on the pure alkyl liquids, the development of the alkyl-dominated surface layer increases significantly with *n*.^32,50^ On the introduction of [C_8_mimF_13_] at a mole fraction of 0.25, an overlayer is formed for all *n*; fluoroalkyl chains are its dominant component for *n* ≤ 8 but a visibly larger area is covered by alkyl chains for *n* = 12.

At a more quantitative level, the ASA analysis can be used to assess the degree of abstractable hydrogen-atom exposure on different surfaces. The results, expressed as fractions (see below), are included in Figures 4 (for C8 only) and 7 (all *n*), where they are compared with the corresponding measures from the RAS-LIF and (more limited) ST data. To carry out this analysis, a choice was necessary about which H-atom types to designate as abstractable. As noted above, we have concluded previously that, due to the only modestly superthermal O-atom translational energy distribution from NO_2_ photolysis,^83^ the H-abstraction is effectively highly selective toward the methylene units on the alkyl chain of the imidazolium cation.^48,50^ On the basis of relative reactivities of related small molecules in the gas phase, confirmed in experiments on related isotopically labeled self-assembled monolayer surfaces,^104,105^ the terminal methyl group is thought to show almost negligible reactivity despite its undoubtedly higher exposure, consistent with its significantly higher C–H bond energy.^84,106^ There is a further important technical uncertainty about the intrinsic reactivities (independent of exposure) of different methylene positions along the alkyl chain in these ILs. We return to this question in the Discussion below, but for the moment, use the error bars in Figures 4 and 7 to indicate the range of results obtained by assuming that the H atoms on either the first two, one, or none of the C atoms nearest the ring in the alkyl chain are excluded from the calculation of accessible reactive secondary hydrogen.

The MD-derived quantities in Figures 4 and 7 represent fractions of the total area of a given surface covered by the accessible reactive secondary-hydrogen atoms and are hence comparable to the other quantities plotted there (This is the appropriate measure because the total probability of those probe O atoms that are directed toward the surface suffering a collision remains at unity, regardless of changes in the surface morphology). The predicted absolute surface area of all atom types per unit geometric area of the slab were found to depend on the liquid. They increased slightly with the increase in fluoro content in the [C_8_mim]_(1–*x*)_[C_8_mimF_13_]_*x*_[Tf_2_N] mixtures (around 10% from *x* = 0 to 1), somewhat more significantly with the alkyl chain length in the pure alkyl liquids (around 19% from *n* = 4 to 12), but only more modestly with chain length in the *x* = 0.25 mixtures (around 4%). Full details are given in Supporting Information 2.6.

Figure 4 shows the MD results for [C_8_mim]_(1–*x*)_[C_8_mimF_13_]_*x*_[Tf_2_N] mixtures, normalized (as in eq 3) to pure [C_8_mim][Tf_2_N]. They show the same qualitative behavior as the RAS-LIF and ST data, with negative deviations of the secondary-hydrogen exposure from linearity ideality. However, they tend to underestimate either of the experimentally observed deviations for low-*x* mixtures. There is closer correspondence when *x* ≥ 0.5.

The variations in secondary-hydrogen exposure with *n* for *x* = 0 and the *x* = 0.25 mixtures are shown in Figure 7. As noted above, there is an (unknown) overall scaling factor relative to the RAS-LIF measurements, so, for the purposes of the comparison in Figure 7a, the MD results have also been normalized to that for pure [C_12_mim][Tf_2_N]. Once again, there is a qualitative agreement with the RAS-LIF results that for all chain lengths, the introduction of 25% [C_8_mimF_13_]^+^ ions leads to a substantial reduction in the alkyl-chain exposure. However, there is a quantitative disagreement about the extent of this reduction and its variation with *n*. This depends on, but is not outweighed by, the assumption of which positions in the alkyl chain are included in the ASA analysis. The apparent agreement between RAS-LIF and MD is somewhat better if the *x* = 0.25 results are considered alone, and the renormalization to experiment is carried out for the *n* = 12 mixture, as shown in Figure 7b. The qualitatively different degrees of dependence of the ratio of *x* = 0.25 to *x* = 0 on *n*, which we emphasize do not depend on any choice of normalization, are highlighted in Figure 7c; as noted above, in the RAS-LIF measurements, this ratio is essentially independent of *n* at around 35%, whereas from the MD ASA analysis, it is in approximate agreement with this for *n* = 4, but increases roughly linearly with *n*. By *n* = 12, the ratio is close to 0.75, which is the stoichiometric result for a mixture containing 25% fluoroalkyl and 75% alkyl chains. Note that an alternative way of expressing this is that the MD results for different *n* would not fall on a single curve in the construction of Figure 4, unlike the RAS-LIF data (We have only shown the *n* = 8 MD data there to avoid clutter.)

## Discussion

The ST measurements, RAS-LIF observations and MD simulations are all in qualitative agreement that fluoroalkyl chains occupy the surface preferentially in mixtures of [C_8_mimF_13_] [Tf_2_N] and [C_*n*_mim][Tf_2_N], at least for *n* ≤ 8. This conclusion follows naturally from the following observations: (i) the negative deviations from linearity in the STs (Figures 3 and 4), given that [C_8_mimF_13_] [Tf_2_N] has a lower ST than [C_8_mim][Tf_2_N]; (ii) the deficits in alkyl-chain exposure relative to linearity observed by RAS-LIF for mixtures of all alkyl chain lengths (Figures 4 and 5), most straightforwardly interpreted as a complementary implicit preference for fluoroalkyl chains; and (iii) the corresponding deficits in the fractions of the surface covered by abstractable secondary hydrogen atoms predicted in the MD simulations (Figures 4 and 7) for chain lengths *n* ≤ 8.

At this qualitative level, our results reinforce previous observations of a generally stronger surface preference for fluoroalkyl chains over alkyl chains.^78−81^ The conformity to the “universal” functional form of the deviation from ideality for other types of IL mixtures noted previously for [C_*n*_mim]_(1–*x*)_[C_8_mimF_13_]_*x*_[Tf_2_N] mixtures with *n* = 8 is also now shown here to extend at least to *n* = 12.^55^

However, there are significant quantitative discrepancies between the ASA analyses of the MD simulations and either our RAS-LIF or ST results, which appear in good agreement with one another where we have data for both (*n* = 8). There are three main aspects:

(A) MD ASA predicts a weaker dependence on chain length, *n*, than RAS-LIF for the pure alkyl liquids (Figure 7a).
(B) MD ASA does not reproduce quantitatively how the ratio of abstractable hydrogen exposure varies with *n* in *x* = 0.25 mixtures (Figure 7c) according to RAS-LIF.
(C) MD ASA also does not reproduce quantitatively the effects of introducing [C_8_mimF_13_][Tf_2_N] at different ratios in [C_8_mim]_(1–*x*)_[C_8_mimF_13_]_*x*_[Tf_2_N] mixtures (Figure 4), found either by RAS-LIF (spanning *n*) or ST (*n* = 8).

Discrepancy (A) has been noted previously.^50^ Discrepancies (B) and (C) are connected and are among the principal additional new observations here. MD ASA appears to support the proposition that longer alkyl chains should compete better with fluorinated chains for surface sites. However, although this may be expected intuitively based on the intrinsically higher surface presence for longer chains in the pure alkyl ILs, it is not borne out in the RAS-LIF experiments here.

The source of discrepancies (A–C) could lie within one, or a combination, of three possibilities:

(1) There is some undetected artifact in the RAS-LIF experiments.
(2) The MD simulations do not give a physically accurate description of the liquid surfaces.
(3) The results of the ASA analysis are not directly comparable with what is measured in the RAS-LIF or ST experiments.

We have no good reason to suspect explanation (1). Note that the discrepancies are obvious even in the raw data; for example, for discrepancy (B), the *x* = 0.25 curve in Figure 5 would have to be more than a factor of 2 or larger for it to agree with the MD ASA prediction for the *x* = 0.25/*x* = 0 ratio. Similar raw data illustrating the other discrepancies are given in Supporting Information 4.1 or in previous publications.^50,55^ None of the discrepancies can, therefore, be a consequence of the way the data are processed to produce relative reactivities and are far beyond the level of statistical uncertainty in the RAS-LIF measurements.

Surface-active contaminants have been found to be a potentially significant issue in related surface-specific experiments on ILs.^107−111^ However, we see no evidence of irreproducibility or irregular trends in the RAS-LIF results; the variations with mixing ratio and with alkyl chain length are all regular and monotonic. The ST data also support the RAS-LIF results for *n* = 8 mixtures. We have, of course, used only one source of fluoro-labeled [C_8_mimF_13_] [Tf_2_N], which could conceivably be the source of discrepancies (B) and (C). However, note that (A) only concerns the *n*-dependence of the RAS-LIF OH yield from the pure alkyl liquids, for which effectively indistinguishable results are obtained here and previously using independent sources of [C_*n*_mim] [Tf_2_N].^48,50^

There is also nothing obvious within the MD simulations themselves to suggest (A–C) can be explained by (2). As described in Supporting Information 2.3–2.5, we are confident that the MD trajectories are fully equilibrated and that the sampling interval is long enough that the snapshots analyzed are uncorrelated. Doubling the number of ion pairs to make slabs which are twice as thick has only minor, predictable effects (see Supporting Information 2.4 and 2.5). Statistical errors based on the variation of ASA results between snapshots are small, even for individual positions on alkyl chains, and much smaller (typically only a few percent) when summed over all secondary hydrogen atoms.

**Figure 12:**
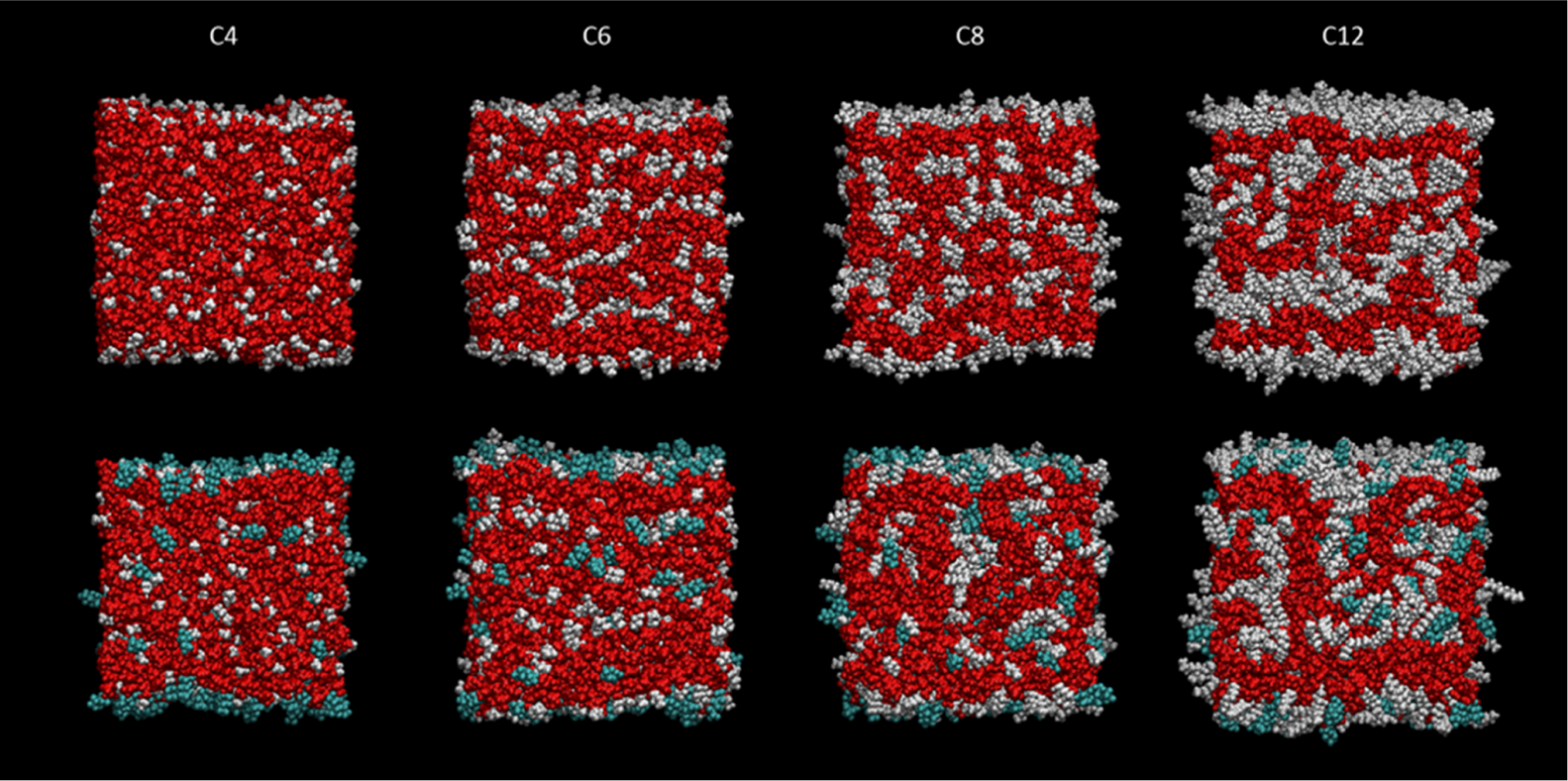
Side view of representative single MD snapshots of pure [C_*n*_mim][Tf_2_N] (upper row) and [C_8_mim]_0.75_ [C_8_mimF_13_]_0.25_ [Tf_2_N] mixtures (lower row) for *n* = 4, 6, 8, and 12. Color scheme as in Figure 8.

As further reassurance, the representative snapshots in Figures 11 and 12 (and see also Supporting Information 2.4 for selected 1600-ion-pair systems) show the expected development of interpenetrating alkyl and polar domains in the bulk and increased alkyl coverage of the surface with the increase in chain length that have already been well-characterized for the parent, nonfluorinated ILs.^32^ For the [C_8_mim]_(1–*x*)_[C_8_mimF_13_]_*x*_[Tf_2_N] mixtures in Figures 9 and 10, the structures are similar except for the visual appearance of two distinct polar domains (alkyl and fluoroalkyl) for intermediate *x*.

**Figure 13:**
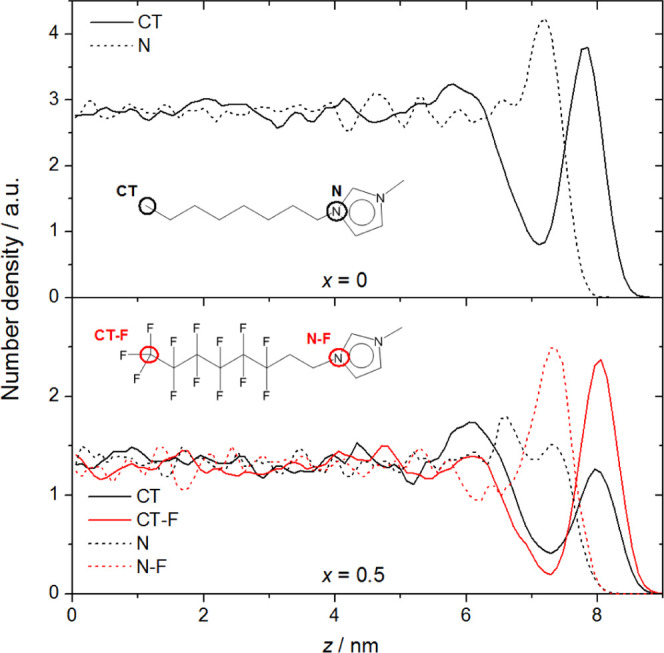
Number density as a function of *z* for the 1600-ion-pair system with *x* = 0 (upper panel) and *x* = 0.5 (lower panel) in the [C_8_mim]_(1–*x*)_[C_8_mimF_13_]_*x*_[Tf_2_N] mixture system at 320 K. The densities of the same atom types for the [C_8_mim]^+^ and [C_8_mimF_13_]^+^ cations are plotted: CT (or CT-F), the terminal carbon atom on the alkyl (or fluoroalkyl) chain and N (or N–F), the ring nitrogen to which the alkyl (or fluoroalkyl) chain is attached. The *z* = 0 position was defined by the center of mass of the slab. Bin widths were ∼0.09 nm, corresponding to 500 slices along the *z*-axis. Averaging is over the equilibrated periods of the MD trajectory, beginning 1 ns (to allow the surface to relax from the previous 500 K heating cycle) into each of the 320 K simulation blocks from 30 to 95 ns.

The disproportionate occupancy of the surface by semiperfluorinated chains is also qualitatively obvious. The nature of these surface layers can be examined more quantitatively by constructing *z*-density profiles (i.e., number density of different atom types in the direction normal to the surface, suitably averaged over both faces of the slab for sufficient length of a trajectory). As an illustrative example of some of the key features, Figure 13 shows the profiles for the terminal methyl carbons (atom-types CT or CT-F, respectively, for alkyl or fluoroalkyl chains) and ring N-atom (atom-types N and N–F, respectively) to which the chains were attached for *x* = 0 and *x* = 0.5 of [C_8_mim]_(1–*x*)_[C_8_mimF_13_]_*x*_[Tf_2_N]. The larger, 1600-ion-pair system is illustrated because it is not subject to minor, undamped oscillations that remain perceptible in the middle of the thinner slabs with 800 ion pairs (see Supporting Information 2.4). The corresponding representative snapshots (final frames of the production runs) are also included for completeness in Supporting Information 2.4.

For *x* = 0, the outer edge of the interface is enriched with CT atoms. The N-atoms occupy a distinct, polar underlayer depleted in alkyl chains, consistent with previous simulations and experimental studies.^32,50,55,112^ There is also the suggestion of a weak, second nonpolar layer around *z* ∼ 6 nm; the depth at which this layer forms, combined with the weak inner shoulder on the N-atom profile, suggest that these are alkyl chains which tend to point inward toward the center of the slab from headgroups in the polar layer. This is confirmed by visual inspection of individual snapshots (see, e.g., the relevant panels of Figure 10 or 12). Such multilayer ordering is consistent with independent MD simulations, confirmed by X-ray scattering and SFG measurements, on related systems.^113−116^

For *x* = 0.5, there is a clear enrichment of [C_8_mimF_13_]^+^ ions at the expense of [C_8_mim]^+^ ions in the outermost layer, as indicated by the corresponding CT-F and CT number densities. The fluoro chains are enriched by a factor of ∼2 relative to their bulk density, whereas the alkyl chains, although still a very clear local maximum, are slightly under-represented relative to the bulk. The immediate polar underlayer is dominated by N–F over N atoms in roughly the same proportions (as required by molecular connectivity). Interestingly, there is a weaker but still obvious secondary polar layer, which is dominated by N over N–F atoms (which are depleted in this region relative to the bulk). Correspondingly, the secondary polar layer at *z* ∼ 6 nm, which is more pronounced than for *x* = 0, contains an excess of CT over CT-F atoms. The breadth of this secondary CT peak suggests that although predominantly inward-pointing, the alkyl chains adopt a wide range of orientations. This is also plausible based on simulations of related systems.^116^

In this absence of any obvious anomalies and barring any undetected errors in the way the MD simulations have been carried out, one remaining possibility within explanation (2) is that the nonpolarizable OPLS-AA/CL&P force fields do not give an accurate representation of the surfaces of these mixed alkyl/fluoro systems. The developers of the OPLS-AA/CL&P framework have recently described its extension to include explicit polarizability using an approach based on Drude dipoles (also known as a “core–shell” model); they have demonstrated its application to the bulk structures of selected ILs.^82,101^ The most significant effects, however, are on ion mobility, producing viscosities and diffusion coefficients in much better agreement with the experiment than classical MD; effects on the structure are more modest. Application to IL surface structures has been very limited so far and is confined to our own treatment of the surface of [C_4_mim][Tf_2_N].^56^ As such, it is not possible to say whether it would significantly alter the surface structures in the more complex alkyl/fluoroalkyl systems here. We simply note, however, that we found very good agreement between RAS-LIF measurements and classical, nonpolarizable MD systems for the surface occupancy of short and long chains in [C_2_mim]_1–*x*_[C_12_mim]_*x*_[Tf_2_N] mixtures.^32^

Our final alternative, explanation (3), concerns the relationship between the results of the ASA analysis of the (now assumed to be physically correct) MD simulations and OH yields in RAS-LIF experiments (now assumed to be free from systematic errors). These two measures are obviously designed to be related, but a priori, they are certainly not identical. Hence, in that sense, differences between RAS-LIF and ASA are not necessarily surprising. However, that is not to say that those differences which can be identified necessarily provide a self-consistent explanation for the observed discrepancies in the results. We consider here two principal aspects: (3)(a) that the OH yield will depend on energetic factors (differential reactivity of different C–H bond types) absent from MD ASA; and (3)(b) that OH yields will separately depend on stereodynamical factors, including impact factors and angles of approach, clearly neglected in a simple ASA analysis.

**Figure 14:**
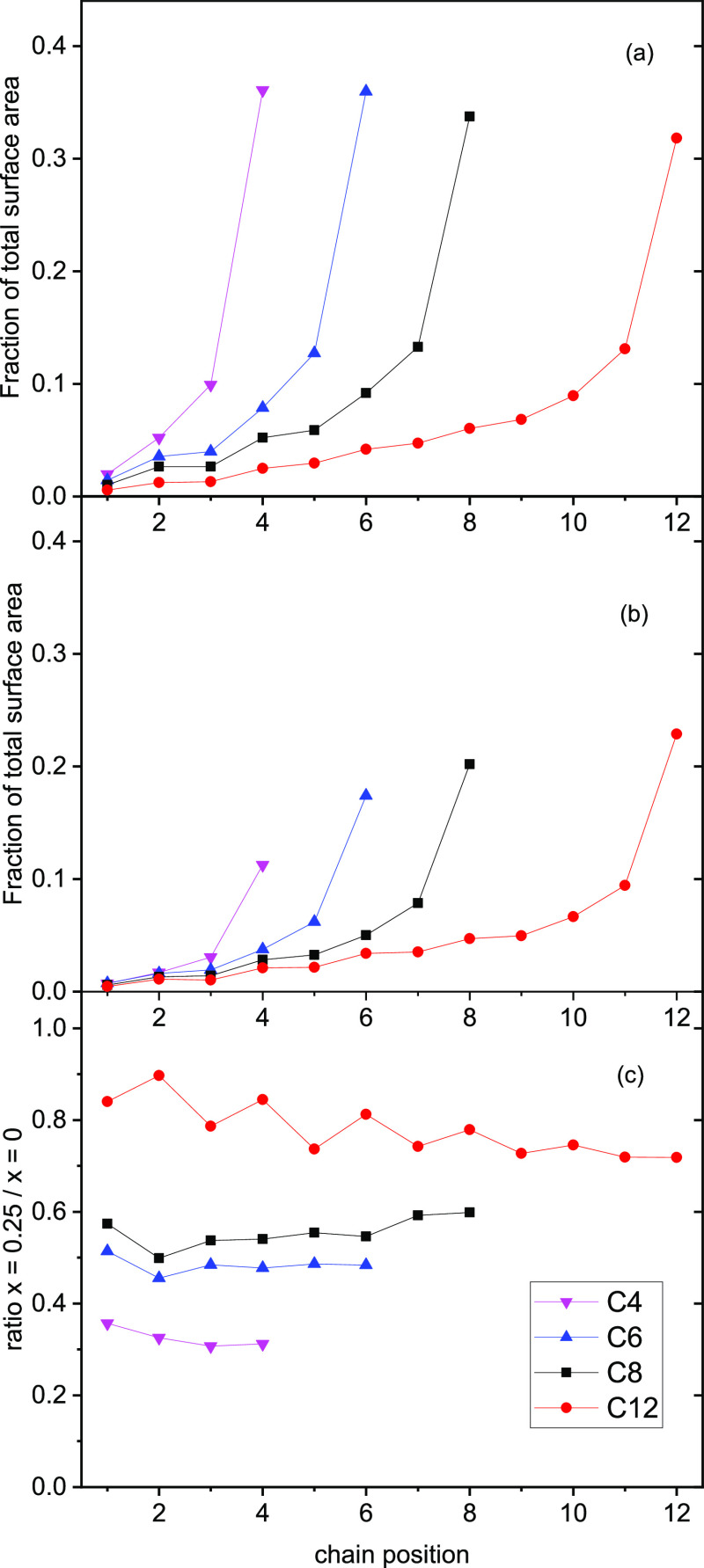
(a) Fraction of the total surface area (sum of all atom types) occupied by the H atoms attached to each C atom in the alkyl chain for pure [C_*n*_mim][Tf_2_N] liquids for *n* = 4–12, as indicated. Chain positions are numbered from the imidazolium ring. Note that the last member of each chain is a methyl group. (b) Corresponding MD ASA fractional areas for the same atom types in [C_*n*_mim]_0.75_[C_8_mimF_13_]_0.25_[Tf_2_N] mixtures. (c) Ratio of the fractional areas of the same atom types in the mixtures, (b), to those in the pure liquids, (a).

Considering the energetic factor (3)(a) first, we have been assuming, as explained above, that the observed OH yield has a negligible contribution from the CH_3_ terminus of the alkyl chains. Although we know this to be a reasonable approximation, based on our previous related work,^50,84,104−106^ it might not be completely reliable. The situation is exacerbated by the relatively large contribution that the terminal CH_3_ group makes to the surface coverage, at least as predicted by MD ASA. This is illustrated in Figure 14a, which shows the fraction of the total surface area occupied by the H atoms at each site in the alkyl chain for the pure [C_*n*_mim][Tf_2_N] liquids for *n* = 4–12. Interestingly, when presented in this form, the fraction of the surface covered by terminal methyl groups is almost independent of chain length. However, as noted above, the total area also increases by ∼19% from *n* = 4 to 12 (see Supporting Information 2.6 for full details) despite a significant absolute reduction in anion exposure, reflecting the increased roughness of the surface as it is dominated by longer alkyl chains. If the CH_3_ groups were to be making a non-negligible contribution to the observed OH yield and should therefore be included to some extent in the MD ASA analysis, note that this would result in an even less steep variation with *n* for the MD ASA results than is currently shown in Figure 7a. The level of this agreement between MD and RAS-LIF would therefore be even poorer. We conclude that any potential CH_3_ reactivity does not in itself provide a resolution of discrepancy (A). As we have noted previously, it does help to rationalize the very significant differences in the *n*-dependence of OH yields from pure alkyl liquids between RAS-LIF using relatively low-energy O(^3^P) atoms from an NO_2_ photolytic source, as here, versus those in related RAS-MS measurements using hyperthermal O(^3^P) atoms.^50^

The issue of differential intrinsic reactivity due to electronic effects is not necessarily confined to differences between primary and secondary H atoms. It is well known from, for example, ARXPS spectroscopy that the electronic environment of the alkyl C atoms is significantly affected by the charge on the imidazolium ring.^110^ The C atoms connected directly to the ring [i.e., the methyl group on N(3) and the first position on the alkyl chain attached to N(1)] were nominally assigned by Lovelock et al. as part of the “ring” (or “C_hetero_”) atoms on the basis of their contribution to the corresponding peak in the XPS spectrum. The remaining members of the alkyl chain form a distinct “C_alkyl_” peak, but the average splitting from the C_hetero_ peak is only fully converged for *n* ≥ 8.

These differences in the electronic environment may also be reflected in higher C–H bond strengths, and correlated higher activation energies for H abstraction for the methylene positions closer to the ring. This is the source of the ambiguity we alluded to above (Results) about whether all the secondary H atoms should be included for comparison with RAS-LIF experiments, or whether some positions closest to the ring should be excluded. However, in practice, this choice makes relatively little difference, as indicated by the error bars in Figure 7a. The reason for this is now obvious from Figure 14a, which shows that the exposure of the −CH_2_– units declines strongly in the direction from the methyl terminus toward the ring. For most chain lengths, it therefore makes little arithmetic difference whether one or both of the first two positions nearest the ring are excluded from the ASA. Naturally, the sensitivity to this choice is largest for *n* = 4. The agreement with the RAS-LIF results in Figure 7a is best, but still systematically poor, when both of the first two positions are excluded.

This may lend some weight to the proposal that electronic effects suppress the reactivity of the −CH_2_– units closest to the ring. We note that in the previous work, we saw essentially no measurable OH signal from [C_2_mim] ILs, consistent with the electronically suppressed reactivity of its sole −CH_2_– unit.^32,48,50^ If so, the other −CH_3_ group attached to the ring at N(3) would have its reactivity doubly suppressed below that of a remote terminal −CH_3_ group. A conceivable resolution of discrepancy (A) might be that the electronic suppression effect propagates further along the chain than the first two positions. This would obviously have most effect for the shorter chains for which the relative discrepancy with the longest C12 chain is the largest. It would be amplified if it extended to the terminus for the shorter chains *and* that these terminal −CH_3_ groups make a non-negligible contribution to OH yield for the longer chains. There would still have to be some suppression as far as *n* = 8 because as Figure 7a shows that the C8/C12 ratio is currently overpredicted. This explanation admittedly remains very speculative, but would in principle be testable through further experiments and supporting ab initio calculations.

A similar electronic effect due to adjacency to the ring, potentially further enhanced by the electron-withdrawing character of the remainder of the fluorinated chain, would also suppress the reactivity of both methylene groups in the −CH_2_CH_2_– linker in [C_8_mimF_13_][Tf_2_N]. However, it is not necessary to invoke this to explain the effective absence of OH yield from pure [C_8_mimF_13_][Tf_2_N] (see Figure 3) because the ASA analysis (see Supporting Information 2.6) implies that these positions are substantially less accessible (by a factor of ∼3.5) than the already minimally exposed equivalent positions in [C_8_mim][Tf_2_N] in Figure 13a. This is consistent with established principles of increased volume and stiffness of fluoro chains relative to their alkyl analogues.

To assess whether discrepancy (B) could be explained by differential energetic effects, that is, 3(a), the fractional ASAs for each chain position in the *x* = 0.25 mixtures are plotted in Figure 14b for comparison with those for the pure alkyl liquids in Figure 14a. Because they must be consistent with Figure 7c, the fractional areas occupied by the alkyl chains in the mixtures necessarily increase with *n* in Figure 14b. What is also apparent, though, from closer inspection of Figure 14a,b is that other than the overall scaling factors, the variations with chain position are of very similar shape for a given *n*. This is confirmed explicitly in Figure 14c, which shows the *x* = 0.25/*x* = 0 ratio for the exposures of each chain position for each *n*. For all chain lengths, the variation with position along the chain is very modest (although interestingly with a clearly systematic odd-even alternation for *n* = 12) and quite tightly distributed around the corresponding overall ratios shown in Figure 7c. This is true also of the terminal methyl groups (which recall do not contribute in Figure 7c). An important conclusion is that although differential reactivities may well be substantial, it is *not possible* that they could explain discrepancy (B). Even changing the weighting of different chain positions substantially in the ASA analysis would not significantly alter the *x* = 0.25/*x* = 0 ratios. No amount of selective inclusion of only specific positions for *n* = 6, 8, or 12 would deliver the constant ratio of ∼0.35 seen for all chain lengths in the RAS-LIF measurements (Figure 7c), which can only be matched via MD ASA for *n* = 4.

A similar analysis (see Supporting Information 2.6) shows that the ratios of surface exposure for different mixing ratios in [C_8_mim]_(1–*x*)_[C_8_mimF_13_]_*x*_[Tf_2_N] mixtures is also only weakly dependent on the position along the C8 alkyl chain. Although the *x*-dependence of the deviations from RAS-LIF experiments in Figure 4 are more subtle than those for the *n*-dependence in Figure 7c, we conclude that it is also unlikely that differential reactivities, 3(a), could explain discrepancy (C).

This leaves (3)(b), stereodynamical factors, as a possible explanation of discrepancies (A–C). The ASA algorithm includes all secondary hydrogen atoms which can be contacted by the probe particle, regardless of the impact parameter or direction of approach.^99^ We had already suggested that such effects might explain discrepancy (A), when it was first noted in previous work.^48,50^ Note carefully, however, that consistent with the discussion above of energetic effects, these will only be satisfactory explanations if the stereodynamical effects vary strongly with the alkyl chain length in pure liquids [to explain (A)] *and* significantly with composition when mixed with fluoroalkyl chains [to explain (B) and (C)], over and above how the exposures assessed by ASA already depend on these variables.

This does not, however, necessarily rule them out. It is believable from inspection of MD snapshots such as those in Figures 9–12, as supported by the limited analyses of chain orientation for different chain lengths that has been carried out,^49,55^ that the outer ends of longer chains tend to lie more parallel to the surface (and perpendicular to the surface normal) than shorter chains. This will increase the probability of the O(^3^P) atom approach close to parallel to the H–C bond axis, which is known to be the strongly preferred geometry from experiments and ab initio potentials for gas-phase reactions of related smaller alkanes.^84,117^ It will also reduce overshadowing of the reactive −CH_2_– positions by the unreactive terminal methyl groups. These effects together might explain discrepancy (A). If either or both effects depend in the correct way on the mixing ratio with fluoroalkyl chains, which would perhaps be at least qualitatively consistent with enhanced overshadowing of alkyl chains by bulky neighboring fluoroalkyl groups, then they might also explain discrepancies (B) and (C). In principle, it would be possible to assess partially whether the implied structural changes are present in the MD simulations by further analysis of MD snapshots. However, this could never be quantitative in the absence of an accurate description of how the reactivity depends on the stereodynamics. Ultimately, this would require a full dynamical scattering calculation. Some progress has been made in this direction using QM/MM methods for reactions of projectiles, including O(^3^P), with liquid surfaces, including model ILs.^118^ However, it is very far from routine, and currently highly questionable whether the relatively low-accuracy level of the ab initio theory necessary to constrain the computational cost would capture the subtle dynamical factors correctly.

Consequently, in the immediate future, it would be more realistic and extremely interesting to compare the current RAS-LIF results for the mixed alkyl/fluoroalkyl systems with alternative experimental measurements of surface composition to establish to what extent the quantitative observations might be method-dependent.

## Conclusions

RAS-LIF measurements on mixtures of variable alkyl chain length 1-alkyl-3-methyl imidazolium-based ILs ([C_*n*_mim][Tf_2_N], *n* = 4, 6, 8, and 12) with a fixed semiperfluorinated C8 chain analogue ([C_8_mimF_13_]_*x*_[Tf_2_N]) imply that the fluorinated chains have a higher surface preference for alkyl chain lengths *n* = 4–12. This is corroborated for *n* = 8 by ST measurements, which agree quantitatively on the under-representation of the alkyl component at the surface.

MD simulations using classical force fields broadly reproduce this effect. However, ASA analyses do not agree quantitatively with the RAS-LIF measurements, or the more-limited ST data, in a number of respects. In particular, the chain-length dependence of neither the RAS-LIF OH yield in the pure alkyl liquids nor its ratio to the yield in a fixed-composition (*x* = 0.25) mixture with [C_8_mimF_13_][Tf_2_N] is predicted correctly by ASA-MD.

An unexpectedly large contribution to the OH yield in RAS-LIF measurements from the terminal methyl groups would not in itself resolve these disagreements and is in any case not supported by independent experimental observations. Potential differential reactivities of methylene groups as a function of their proximity to the imidazolium ring are also not able to explain all the discrepancies. The remaining possibility, within the assumption that the MD simulations are physically correct, is that subtle stereochemical effects affect the OH yield in a way that depends both on alkyl chain length and on the influence of fluorinated chains on the mixtures.

Alternatively, the classical force-fields used in the MD simulations may not be able to capture the structure of the liquid surface correctly. It would be interesting to test this using more sophisticated polarizable force fields and also to examine these intriguing materials through alternative surface-sensitive experimental methods. If they are to be used for future tailored applications in which uptake at, and transport of, gases through the interface is manipulated, or accessibility of solvated molecules is controlled, a clear understanding of the principles that affect their surface structures will be required.

## Supporting Information Available

Commercial IL characteristics; MD simulations; ST measurements; RAS-LIF OH appearance profiles; RAS-LIF rotational distributions; and integrated RAS-LIF OH number densities (PDF)
