# Supplementary material for: Surface Structure of Alkyl/Fluoroalkylimidazolium Ionic–Liquid Mixtures
Source: J Phys Chem B. 2022 Feb 28;126(9):1962–79. doi: 10.1021/acs.jpcb.1c10460 (PMC9007465; doi:10.1021/acs.jpcb.1c10460)
Supplement: Supplementary file 1 — jp1c10460_si_001.pdf [file jp1c10460_si_001.pdf]

Surface Structure of Alkyl/Fluoroalkylimidazolium Ionic-Liquid Mixtures

Simon M. Purcell,[†] Paul D. Lane,[†] Lucía D'Andrea,[§] Naomi S. Elstone,[§] Duncan W. Bruce,[§ iD] John M. Slattery,[§, iD] Eric J. Smoll, Jr.,[‡] Stuart J. Greaves,[† iD] Matthew L. Costen,[† iD] Timothy K. Minton,[⁑,* iD] and Kenneth G. McKendrick[†,* iD]*

[†]Institute of Chemical Sciences, School of Engineering and Physical Sciences, Heriot-Watt University, Edinburgh, EH14 4AS, United Kingdom.

[‡]Department of Chemistry and Biochemistry, Montana State University, Bozeman, Montana 59717, United States.

[⁑]Ann and H.J. Smead Department of Aerospace Engineering Sciences, University of Colorado Boulder, Boulder, CO 80303, USA

[§]Department of Chemistry, University of York, Heslington, York YO10 5DD, United Kingdom.

[iD] ORCID ids:
Paul D. Lane 0000-0003-4819-7714
Naomi S. Elstone 0000-0003-0176-1445
Duncan W. Bruce 0000-0002-1365-2222
John M. Slattery 0000-0001-6491-8302
Stuart J. Greaves 0000-0002-5238-7150
Matthew L. Costen 0000-0002-6491-9812
Timothy K. Minton 0000-0003-4577-7879
Kenneth G. McKendrick 0000-0001-8979-2195

Contents

1. Materials 3

1.1. Commercial ionic liquid characteristics 3

2. Molecular Dynamics Simulations 4

2.1. Run Parameters and Protocols 4

2.2. ASA Threshold Dependence 9

2.3. Block averaging 10

2.4. System size comparison: density 14

2.5. System size and run time comparison 16

2.6. MD Areas..18

3. Surface tension measurements 24

3.1. Surface tension results $[C_8mim]_{(1-x)}[C_8mimF_{13}]_x[NTf_2]$ 24

4. RAS-LIF OH appearance profiles 25

4.1. Number density profiles for $[C_6mim]_{(1-x)}[C_8mimF_{13}]_x[NTf_2]$ and $[C_4mim]_{(1-x)}[C_8mimF_{13}]_x[NTf_2]$ 25

5. RAS-LIF rotational distributions 26

5.1. $[C_{12}mim]_{0.5}[C_8mimF_{13}]_{0.5}[NTf_2]$ and $[C_{12}mim][NTf_2]$ rotationally resolved spectra 26

5.2. Rotational analysis 26

6. Integrated RAS-LIF OH number densities to derive reactivity 28

6.1. $[C_{12}mim]_{(1-x)}[C_8mimF_{13}]_x[NTf_2]$ mixtures..28

6.2. $[C_8mim]_{(1-x)}[C_8mimF_{13}]_x[NTf_2]$ mixtures 29

6.3. $[C_6mim]_{(1-x)}[C_8mimF_{13}]_x[NTf_2]$ 30

6.4. $[C_4mim]_{(1-x)}[C_8mimF_{13}]_x[NTf_2]$ 30

7. Supplementary references 31

1. Materials

1.1. Commercial ionic liquid characteristics

While most experiments were performed with ILs prepared in house (see main text), we also performed repeat RAS-LIF measurements at the end of the study on two ILs, [C_8mim][Tf_2N] and [C_{12}mim][Tf_2N], supplied by by IoLiTec (Germany) and used as received. Table S1 shows the manufacturer's characterisation data for these liquids.

Liquid	Purity	[H_2O]	[Halides]
[C_8mim][NTf_2]	>99%	53 ppm	<100 ppm
[C_{12}mim][NTf_2]	>99.9%	57 ppm	<100 ppm

Table S1: Ionic liquid purity information for commercial samples.

2. Molecular Dynamics Simulations

2.1. Run Parameters and Protocols

A 2 fs time step was used in conjunction with the leap frog integrator. Lengths of all bonds to H atoms were constrained. Shifted potentials with energy and pressure corrections were used with 1.5 nm cut-offs for the van der Waals and Coulomb potentials. The long-range electrostatic potential was calculated using particle-mesh Ewald with an order of 4 and minimum grid spacing of 0.12 nm. The data were saved every 2 ps and 4 ps for the small (800 ion pairs) and large (1600 ion pairs; n = 8, x = 0 and 0.5 only) simulations respectively.

The composition and dimensions of the systems studied are listed in Table S2. Each liquid was simulated following one of three protocols. The protocols varied slightly in run length for the bulk equilibration phase and in the slab heating and cooling cycles for the slab. Table S3 lists the protocols and the liquids to which they were applied. Tables S4 to S6 show the simulation parameters. The time constants for the barostat and thermostats are represented by τ_p and τ_t respectively.

x	$[C_n mim]^+$	$[C_8 mim F_{13}]^+$	$[NTf_2]^+$	Atoms	Initial box size / nm	Slab size[a] / nm	Liquid ID number
				n = 8			
0	800	0	800	41600	$x = y = 9.0$	x = y = 7.934	1
0.1	720	80	800	41600	$x = y = 8.5$	x = y = 7.785	2
0.25	600	200	800	41600	$x = y = 8.5$	x = y = 7.831	3
0.5	400	400	800	41600	$x = y = 9.0$	x = y = 7.934	4
0.75	200	600	800	41600	x = y = 8.5	x = y = 8.031	5
1	0	800	800	41600	x = y = 9.0	x = y = 8.121	6
				Large system, n = 8			
0	1600	0	1600	83200	$x = y = 8.5$, $z = 17.0$	$x = y = 7.746$, $z = 46.478$	7
0.5	800	800	1600	83200	$x = y = 9.0$,	$x = y = 7.937$,	8

					z = 18.0	z = 47.621	
n = 12							
0	800	0	800	51200	$x = y = z =$ 9.0	$x = y$ = 8.219	9
0.25	600	200	800	48800	$x = y = z =$ 9.0	$x = y$ = 8.186	10
n = 6							
0	800	0	800	36800	$x = y = z =$ 7.8	$x = y$ = 7.747	11
0.25	600	200	800	38000	$x = y = z =$ 8.1	$x = y$ = 7.654	12
n = 4							
0	800	0	800	32000	$x = y = z$ =7.5	$x = y$ = 7.194	13
0.25	600	200	800	34400	$x = y = z =$ 8.1	$x = y$ = 7.446	14

Table S2: System sizes for MD simulations. [a]z dimension of simulation cell can be obtained by multiplying x or y by 3.

Protocol followed	Liquid ID
1	1, 4, 6
2	7, 8
3	2, 3, 5, 9 - 14

Table S3: Protocol followed for each liquid.

Run	Barostat	Pressure / bar	τ_p / ps	Thermostat	τ_t / ps	Temp / K	Run length / ns	Total run time / ns
Bulk								
1	Berendsen	1.0	2.0	v-rescale	0.5	500	0.2	-

2	Parrinello-Rahman	1.0	2.0	v-rescale	0.5	320	2.0	-
Slabs								
1				v-rescale	0.5	320	5	5
2				v-rescale	0.5	500	5	10
3				v-rescale	0.5	320	5	15
4				v-rescale	0.5	500	5	20
5				v-rescale	0.5	320	5	25
6				v-rescale	0.5	500	5	30
7				v-rescale	0.5	320	5	35
8				v-rescale	0.5	500	5	40
9				v-rescale	0.5	320	5	45
10				v-rescale	0.5	500	5	50
11				v-rescale	0.5	320	10	60
12				v-rescale	0.5	500	5	65
13				v-rescale	0.5	320	5	70
14				v-rescale	0.5	500	5	75
15				v-rescale	0.5	320	5	80
16				v-rescale	0.5	500	5	85
17				v-rescale	0.5	320	10	95

Table S4: Protocol 1.

Run	Barostat	Pressure / bar	τ_p / ps	Thermostat	τ_t / ps	Temp / K	Run length / ns	Total run time / ns
Bulk								
1	Berendsen	1.0	2.0	v-rescale	0.5	500	0.5	-
2	Parrinello-Rahman	1.0	2.0	v-rescale	0.5	320	4.0	-

Slab								
1				v-rescale	0.5	320	5	5
2				v-rescale	0.5	500	5	10
3				v-rescale	0.5	320	5	15
4				v-rescale	0.5	500	5	20
5				v-rescale	0.5	320	5	25
6				v-rescale	0.5	500	5	30
7				v-rescale	0.5	320	5	35
8				v-rescale	0.5	500	5	40
9				v-rescale	0.5	320	5	45
10				v-rescale	0.5	500	5	50
11				v-rescale	0.5	320	10	60
12				v-rescale	0.5	500	5	65
13				v-rescale	0.5	320	5	70
14				v-rescale	0.5	500	5	75
15				v-rescale	0.5	320	5	80
16				v-rescale	0.5	500	5	85
17				v-rescale	0.5	320	10	95

Table S5: Protocol 2.

Run	Barostat	Pressure / bar	τ_p / ps	Thermostat	τ_t / ps	Temp / K	Run length / ns	Total run time / ns
Bulk								
1	Berendsen	1.0	2.0	v-rescale	0.5	500	0.5	
2	Parrinello-Rahman	1.0	2.0	v-rescale	0.5	320	4.0	
Slab								
1				v-rescale	0.5	320	5	5
2				v-rescale	0.5	500	5	10

3				v-rescale	0.5	320	5	15
4				v-rescale	0.5	500	5	20
5				v-rescale	0.5	320	5	25
6				v-rescale	0.5	500	5	30
7				v-rescale	0.5	320	5	35
8				v-rescale	0.5	500	5	40
9				v-rescale	0.5	320	5	45
10				v-rescale	0.5	500	5	50
11				v-rescale	0.5	320	5	55
12				v-rescale	0.5	500	5	60
13				v-rescale	0.5	320	5	65
14				v-rescale	0.5	500	5	70
15				v-rescale	0.5	320	5	75
16				v-rescale	0.5	500	5	80
17				v-rescale	0.5	320	10	90

Table S6: Protocol 3.

The experimental bulk densities are shown in table S7.

Liquid	Exp density / g cm^{-3}	Simulation / g cm^{-3}	Error / %
n = 4	1.415	1.491	5.4
n = 6	1.344	1.420	5.6
n = 8	1.292	1.361	5.3
n = 12	1.226	1.273	3.8

Table S7: Extrapolated densities from experimental measurements compared against those calculated from MD here.

2.2. ASA Threshold Dependence

The ASA method reports the surface area of each atom in the simulation frame. The algorithm was used with a probe-particle radius of 0.15 nm, corresponding to the van der Waals radius of an oxygen atom, and 10,000 dots for the resolution.

A small number of false atom counts from bulk voids were also detected by the ASA method. For the purposes of carrying out preliminary tests of equilibration and convergence, etc, the false counts were eliminated by applying a surface area threshold, T_{area}, before a hydrogen atom could be counted as being at the vacuum-liquid interface. An appropriate value was selected by examining the x = 0.5 to x = 1 ratio of secondary hydrogen atoms, (where the surface count of secondary hydrogens was averaged over the simulation run starting from 1 ns) as a function of threshold area, for the C8 system. This is shown in figure S1 for different simulation run times. At low T_{area}, there is a slow decline in the ratio until between 0.15 – 0.20 nm^2, where the ratio does not vary significantly. With larger values of T_{area}, the ratio can diverge causing an initial increase in the ratio before reaching zero as T_{area} exceeds the area that a fully exposed atom presents to the probe particle. This analysis was also applied to the larger 1600 ion pair system, and to the x = 0.25 C4 mixture, with similar results. Therefore, a threshold value of 0.19 nm^2 was arbitrarily chosen when calculating the surface H-atom count for equilibration tests between runs.

For the subsequent, more-detailed quantitative comparison of the exposures of different atom types, including different positions along alkyl chains, the total exposed area of each type was summed, without applying any threshold. Although, as noted above, this admits a very minor contribution from atoms which are not truly at the surface, it avoids more significant systematic distortions resulting from differential sensitivities of different atom types to the choice of the threshold area.

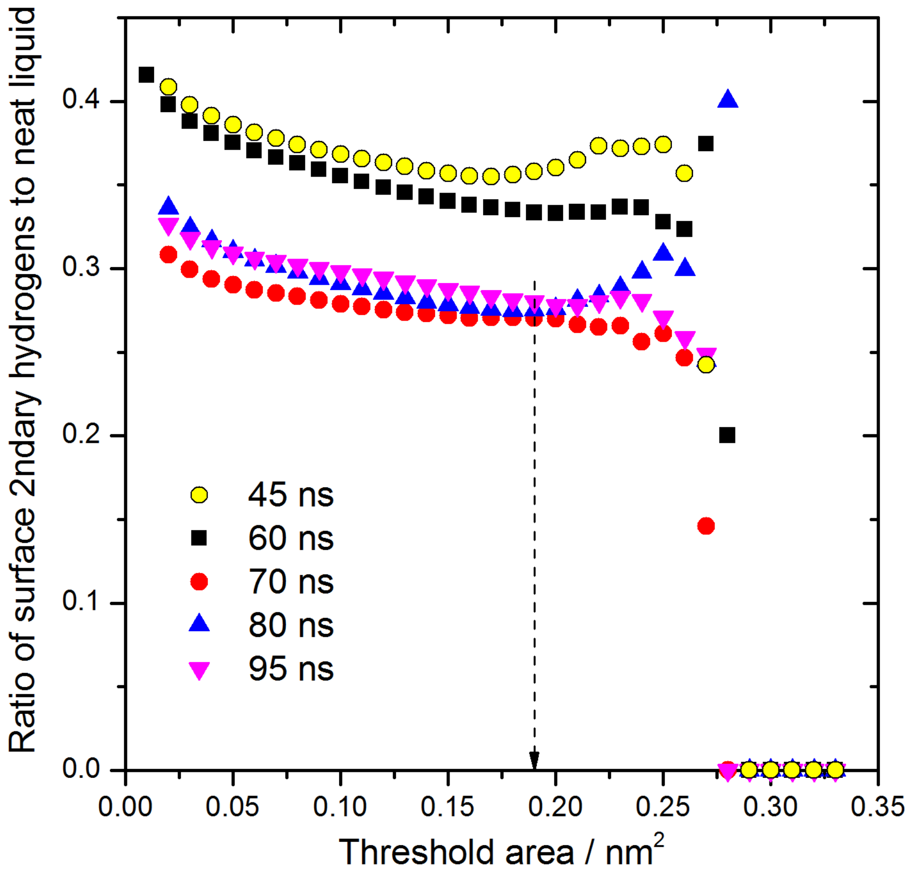

Figure S1: Ratio of average number of surface secondary hydrogens to that of the neat liquid as the threshold is varied for $[C_8mim]_{(1-x)}[C_8mimF_{13}]_x[NTf_2]$ with x = 0.5. Several runs with the total simulation runtime being are shown. The dashed arrow indicates the chosen threshold value of 0.19 nm^2.

2.3. Block averaging

A block analysis[1] was used to determine the relaxation times over several 320 K liquid runs. The ASA values (thresholded secondary-H atom count) were calculated from 1 ns after the start of the run to allow the surface to relax from 500 K. The simulation run is divided into blocks of variable length, b, so that the total number of blocks in a simulation run is n_b, alternatively, $n_b = T/b$, where T is the total length of the simulation measured in saved data points. The mean of each block, $\langle A \rangle_b$, is calculated according to

$$\langle A\rangle_b = \frac{1}{b}\sum_{i=1}^{b} A_i \quad (Eqn\ S1)$$

where A_i is the ASA value from a single frame, *i*, in the simulation and $\langle A_b\rangle$ is the average for a single block of length, *b*. The variance of the mean for each block from the overall mean is calculated, and then averaged according to

$$\sigma^2(\langle A_b\rangle) = \frac{1}{n_b}\sum_{b=1}^{n_b} (\langle A\rangle_b - \langle A\rangle_T)^2 \quad (Eqn\ S2)$$

where $\sigma^2(\langle A_b\rangle)$ is the averaged variance of the blocks for a given block length, *b*, $\langle A\rangle_T$ is the mean ASA value over the all of the frames in the simulation (excluding the first 1 ns). The statistical inefficiency was calculated as

$$s = \lim_{b\to\infty} \frac{b\sigma^2(\langle A_b\rangle)}{\sigma^2(A)_T} \quad (Eqn\ S3)$$

where $\sigma^2(A)_T$ is the variance over all simulation frames. Figures S2 to S4 show representative graphs of *s*, plotted as a function of step size, for several different systems. Generally, between 0.3-0.6 ns, the graphs show a roll off, indicating the data sampled with this period is becoming uncorrelated. The sampling period of 0.4 ns was chosen as this gave a good compromise between having enough frames to average over from each run and reducing the correlation between frames.

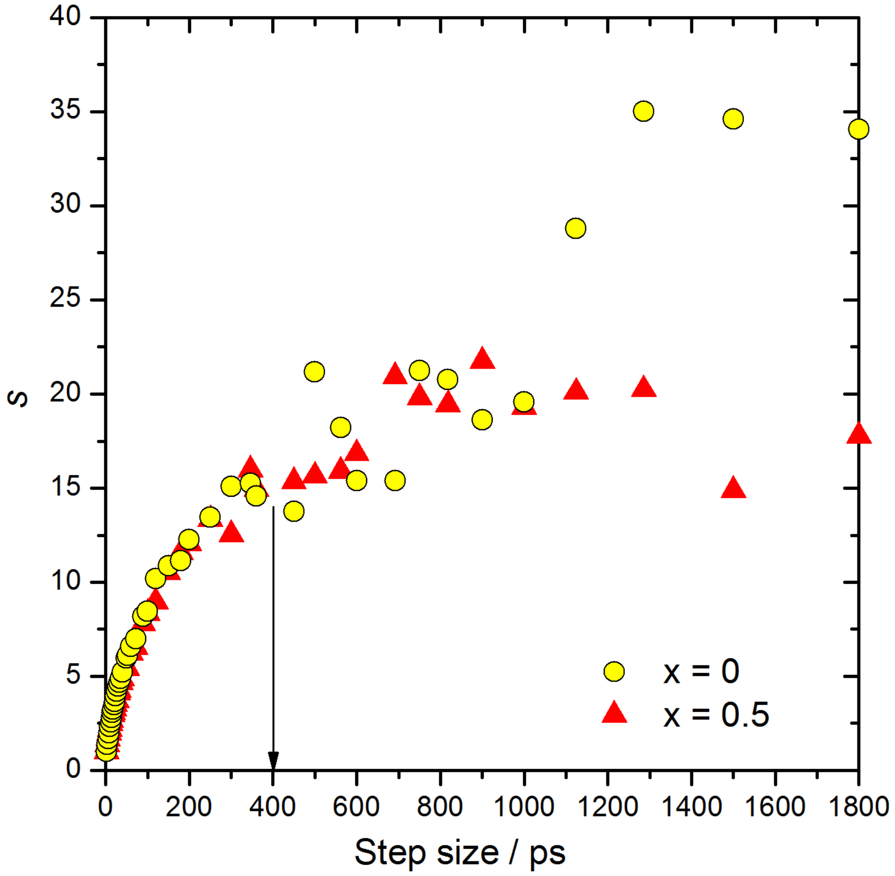

Figure S2: Block analysis applied to run 11 (10 ns length) for $[C_8mim]_x[C_8mimF_{13}]_{(1-x)}[NTf_2]$ with 800 ion pairs. The arrow shows the selected sampling period of 0.4 ns, occurring near the roll-off for *s*.

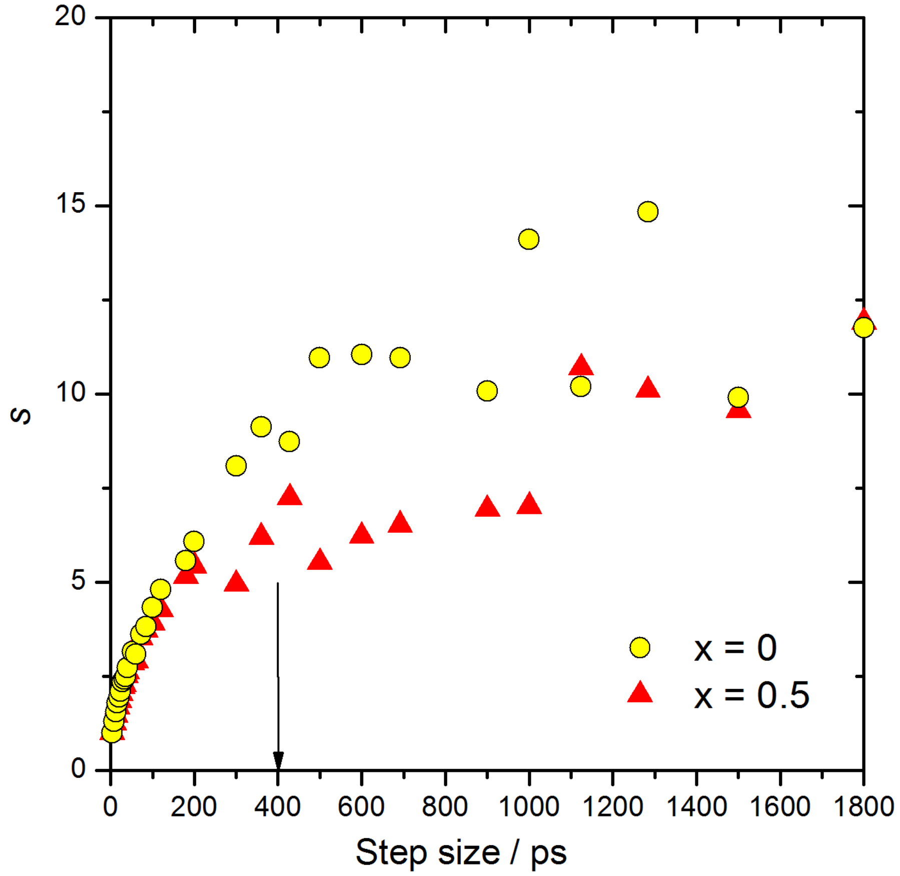

Figure S3: Block analysis applied to run 17 (10 ns length) for the large system, $[C_8mim]_x[C_8mimF_{13}]_{(1-x)}[NTf_2]$, with 1600 ion pairs. The arrow shows the 0.4 ns sampling period.

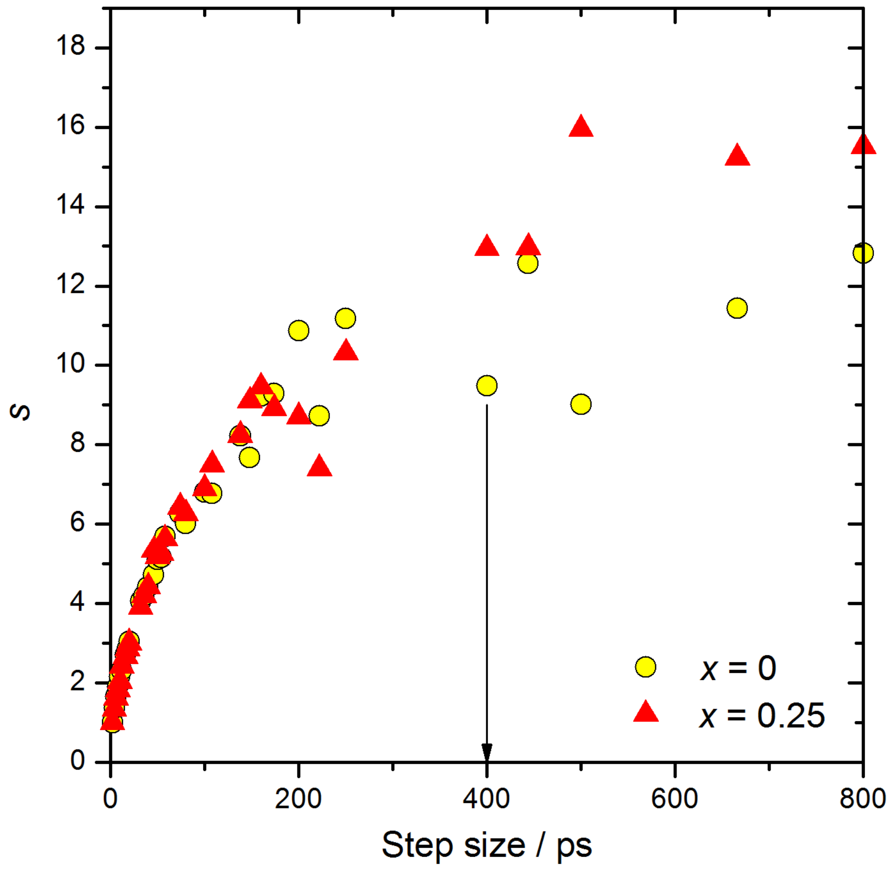

Figure S4: Block analysis applied to run 7 (5 ns length) for $[C_6mim]_x[C_8mimF_{13}]_{(1-x)}[NTf_2]$. The arrow shows the 0.4 ns sampling period.

The convergence of the z-density profiles was monitored for the *x* = 0 and *x* = 1 for the 800 and 1600 ion pair system and it was found from 35 ns, the outer atomic layer did not change significantly. (Further, more extensive tests of convergence, which gave similar results, are given in S2.5 below.) The larger systems reach constant densities at lower values of *z*, corresponding to the middle of the slab, whereas the smaller system still shows some weak undamped density oscillations towards *z* = 0, as shown in Figure S5.

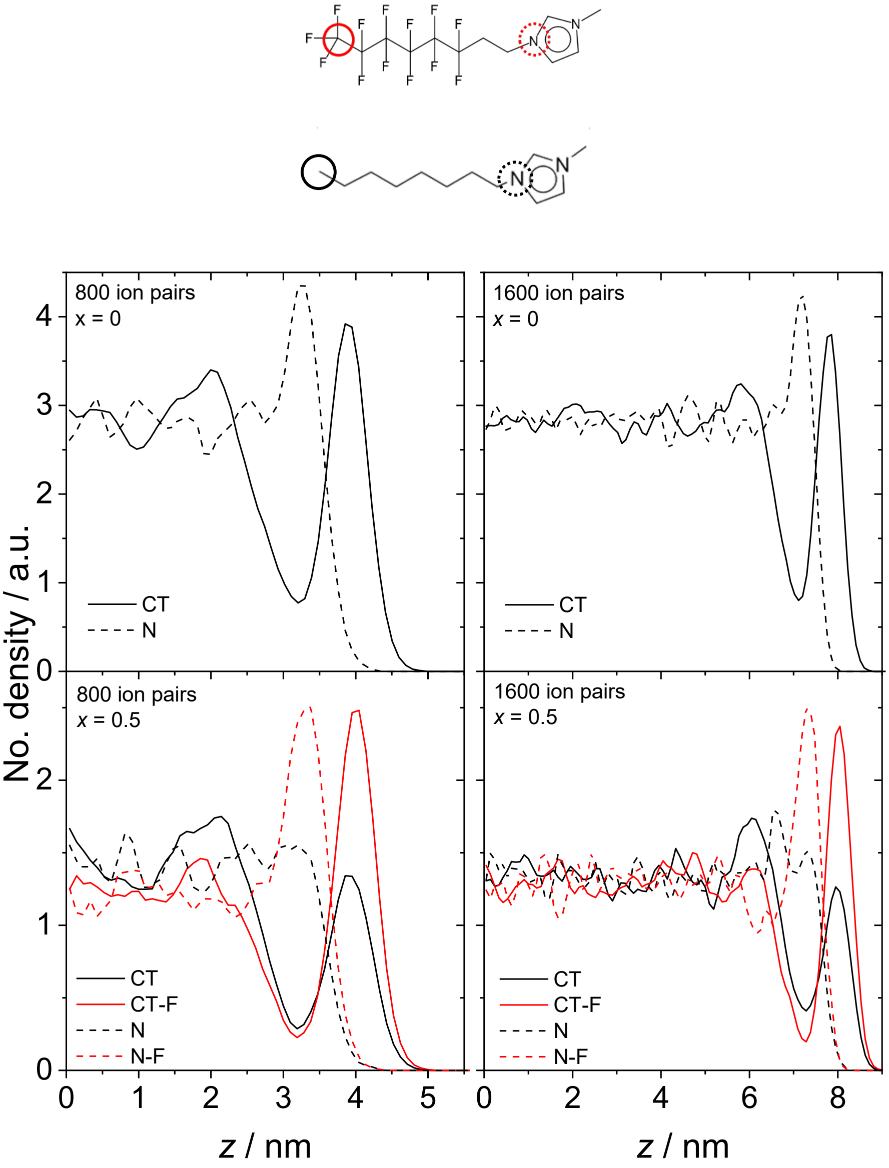

Figure S5: Density profiles for the two different system sizes, 800 ion pairs (top row), 1600 ion pairs (bottom row) for $[C_8mim]_x[C_8mimF_{13}]_{(1-x)}[NTf_2]$ with $x = 0$ and $x = 0.5$. The atom types are indicated by lines as shown; they are the same as Figure 13 in the main article. Averages from 35 to 90 ns run time.

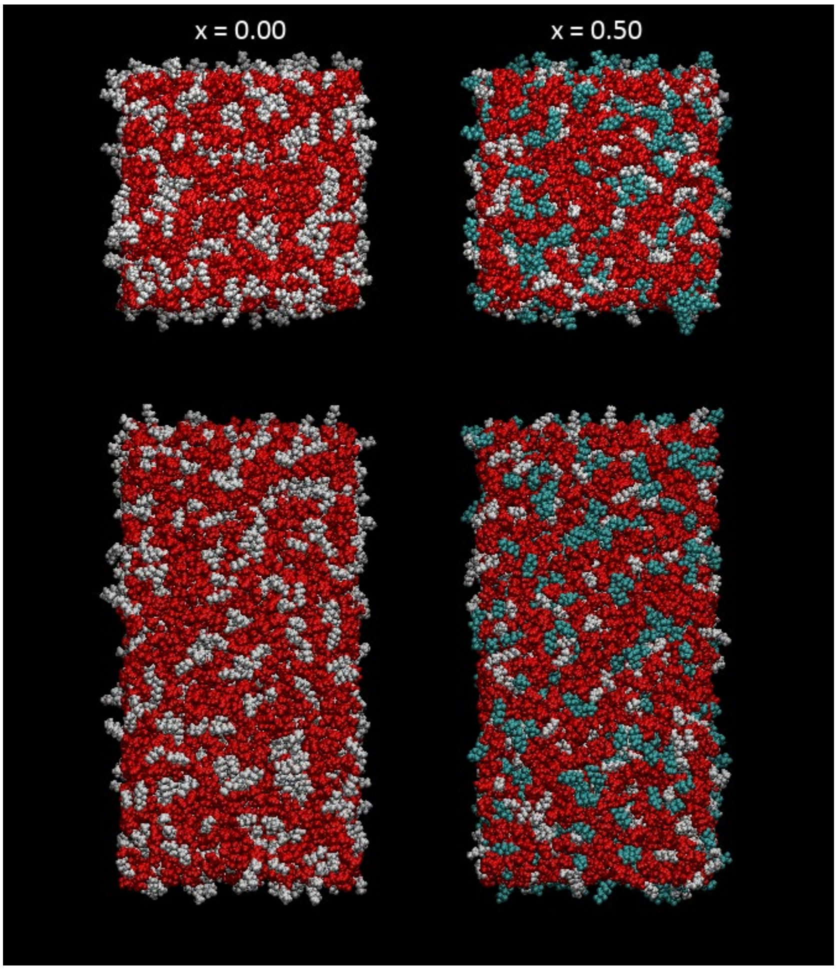

Figure S6: Cation density for [C_8mim] and [C_8mimF_{13}] in the [C_8mim]$_{0.5}$[C_8mimF_{13}]$_{0.5}$[NTf_2] mixture. Top frames= top view; bottom frames = side view. The color scheme is as defined in the main text (Figure 8).

The runtime dependence of the exposed secondary-hydrogen exposed atom count, normalised to that for pure [C_8mim] [NTf_2], for the large and small [C_8mim]$_{0.5}$[C_8mimF_{13}]$_{0.5}$[NTf_2] systems is shown in figure S7. We conclude that there are no significant differences between the large and small systems. Also shown in Figure S8 is the runtime dependence for a selection of other liquid mixtures of different composition and chain length, as indicated. In all cases, and as in Fig. S7, results are stable generally, and with

confidence beyond ~45 ns. This is the basis for choosing to analyse surface composition beyond this time to obtain average quantities, as stated in the main text. There are also no significant differences between averaging every frame ('total mean'), or (much more efficient) sampling every 400 ps, as derived in Section S2.3 and adopted for the analysis of production runs.

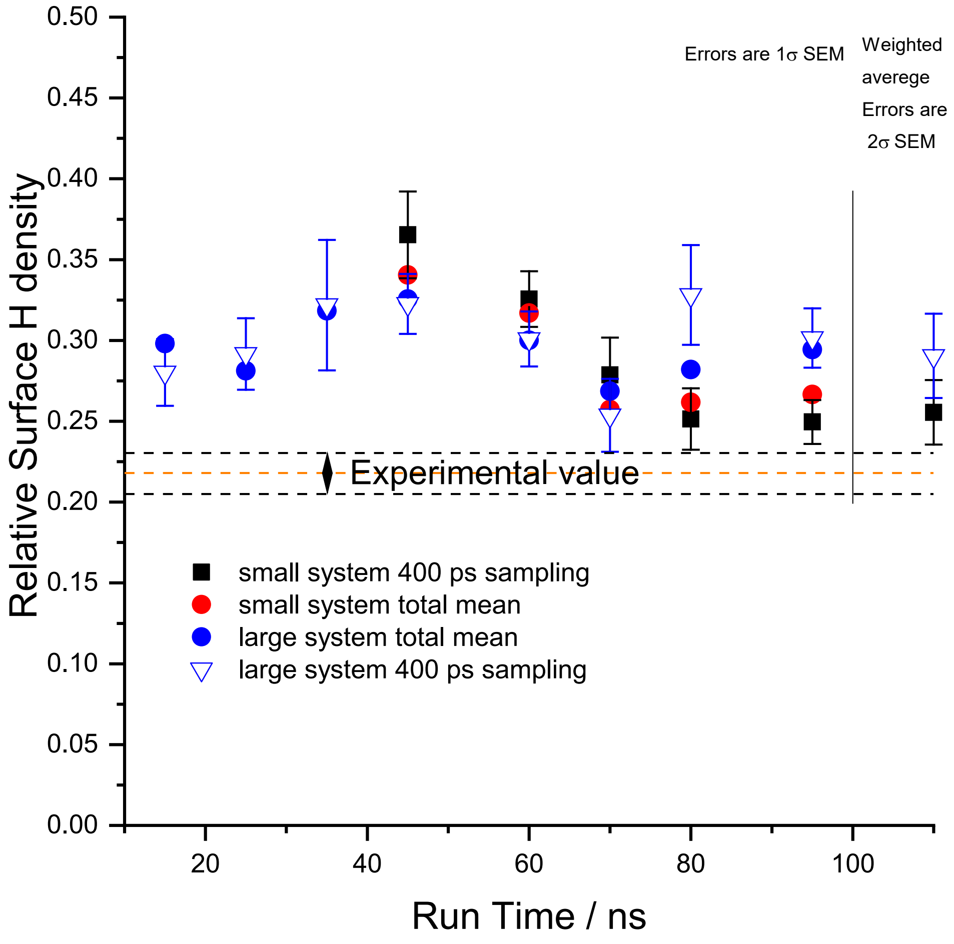

Figure S7: Relative surface H-density as a function of run time for the large and small system for $[C_8mim]_{0.5}[C_8mimF_{13}]_{0.5}[NTf_2]$. Sampling intervals as indicated.

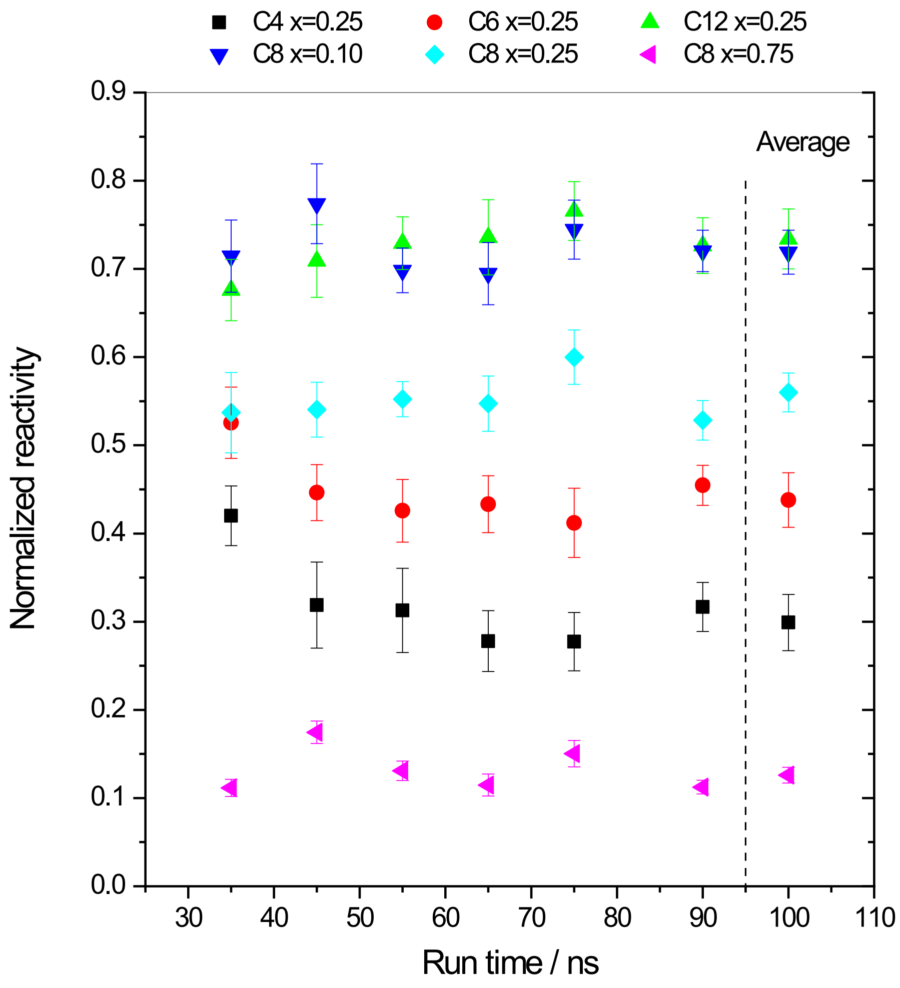

Figure S8: Normalized reactivity (as defined in the main text) as a function of run time for a selection of liquid mixtures, with the compositions indicated. Errors are 1σ SEM.

2.6. MD Areas

ASA-determined areas for different liquids are tabulated below.

	ASA Total surface area (all atoms) / nm^2	Geometric Slab area (per face) / nm^2	Ratio surface/2 slab faces	Ratio Normalised to C8mimF
C8mim	213.1406	59.9234	1.7784	0.9095
C8mim10	218.9873	60.6070	1.8066	0.9239
C8mim25	224.4879	61.3299	1.8301	0.9359
C8mim50	236.6350	62.9541	1.8794	0.9611
C8mim75	250.0592	64.4910	1.9387	0.9914
C8mimF	257.8911	65.9440	1.9553	1.0000

Table S8: ASA measured areas and slab areas for C_8mimX liquids. (Mole fraction of fluoro component = x = X / 100 in $[C_8mim]_{(1-x)}[C_8mimF_{13}]_x[NTf_2]$ mixtures.)

	ASA Total surface area (all atoms) / nm^2	Geometric Slab area (per face) / nm^2	Ratio surface/2 slab faces	Ratio Normalised to C12mim
C4mim	160.7555	51.7598	1.5529	0.8436
C6mim	189.3527	55.8547	1.6950	0.9208
C8mim	213.1406	59.9233	1.7784	0.9662
C12mim	248.6578	67.5439	1.8407	1.0000

Table S9: ASA measured areas and slab areas for pure Cnmim (=$[C_n mim]$ $[NTf_2]$) liquids.

	ASA Total surface area (all atoms) / nm^2	Geometric Slab area (per face) / nm^2	Ratio surface/2 slab faces	Ratio Normalised to C12mim25

C4mim25	199.4408	55.4373	1.7987	0.9626
C6mim25	209.9967	58.5823	1.7923	0.9591
C8mim25	224.4879	61.3299	1.8302	0.9793
C12mim25	250.4270	67.0054	1.8687	1.0000

Table S10: ASA measured areas and slab areas for Cnmim25 (= $[C_8mim]_{0.75}[C_8mimF_{13}]_{0.25}[NTf_2]$) mixtures.

	Surface area / nm^2				Ratio		
X	Fluoro	Alkyl	Anion	Total	Fluoro	Alkyl	Anion
0.00	0.0000	171.8607	41.2798	213.1406	0.000	0.806	0.194
0.10	47.6554	136.6423	34.6542	218.9520	0.218	0.624	0.158
0.25	87.1703	104.9887	32.2882	224.4472	0.388	0.468	0.144
0.50	151.8677	56.3706	28.3967	236.6350	0.642	0.238	0.120
0.75	196.0917	25.9018	28.0129	250.0064	0.784	0.104	0.112
1.00	231.3420	0.0000	26.4968	257.8388	0.897	0.000	0.103

Table S11: Total surface areas and fraction of surface occupied by fluoroalkyl cation, alkyl cation and anion for $[C_8mim]_{(1-x)}[C_8mimF_{13}]_x[NTf_2]$.

	Surface area / nm^2			Ratio	
N	Alkyl	Anion	Total	Alkyl	Anion
4	99.4597	61.2739	160.7336	0.619	0.381
6	139.3525	49.9664	189.3189	0.736	0.264
8	171.8607	41.2798	213.1406	0.806	0.194
12	224.8046	23.8099	248.6145	0.9042	0.0958

Table S12: Total surface areas and fraction of surface occupied by fluoroalkyl cation, alkyl cation and anion for $[C_nmim][NTf_2]$.

	Surface area / nm^2	Ratio

N	Fluoro	Alkyl	Anion	Total	Fluoro	Alkyl	Anion
4	122.2086	39.4535	37.7501	199.4123	0.613	0.198	0.189
6	97.6064	75.2617	37.0946	209.9627	0.465	0.358	0.177
8	87.1703	104.9887	32.2882	224.4472	0.388	0.468	0.144
12	58.6466	167.0259	24.6990	250.3715	0.234	0.667	0.099

Table S13: Total surface areas and fraction of surface occupied by fluoroalkyl cation, alkyl cation and anion for $[C_nmim]_{(0.75)}[C_8mimF_{13}]_{0.25}[NTf_2]$.

	Alkyl								Fluoro
x	C1 H’s	C2 H’s	C3 H’s	C4 H’s	C5 H’s	C6 H’s	C7 H’s	C8 H’s (CH_3)	C1&C2 H’s
0.00	0.0104	0.0265	0.0264	0.0522	0.0589	0.0916	0.1328	0.3374	0.0000
0.10	0.0082	0.0191	0.0197	0.0380	0.0437	0.0693	0.1035	0.2682	0.0031
0.25	0.0060	0.0132	0.0142	0.0283	0.0327	0.0501	0.0787	0.2021	0.0046
0.50	0.0032	0.0075	0.0085	0.0144	0.0165	0.0253	0.0387	0.1010	0.0079
0.75	0.0017	0.0039	0.0034	0.0066	0.0067	0.0110	0.0159	0.0419	0.0103
1.00	-	-	-	-	-	-	-	-	0.0106

Table S14: Fraction of solvent accessible area of hydrogen atoms by chain position for $[C_8mim]_{(1-x)}[C_8mimF_{13}]_x[NTf_2]$

n	C1 H’s	C2 H’s	C3 H’s	C4 H’s	C5 H’s	C6 H’s	C7 H’s	C8 H’s	C9 H’s	C10 H’s	C11 H’s	C12 H’s
4	0.0198	0.0523	0.0992	0.3609	-	-	-	-	-	-	-	-
6	0.0144	0.0354	0.0399	0.0787	0.1272	0.3596	-	-	-	-	-	-
8	0.0103	0.0265	0.0265	0.0523	0.0589	0.0916	0.1329	0.3375	-	-	-	-

12	0.0056	0.0124	0.0132	0.0249	0.0294	0.0418	0.0474	0.0603	0.0683	0.0893	0.1312	0.3183

Table S15: Fraction of solvent-accessible area of hydrogen atoms by chain position for pure [$C_n$mim][NTf_2]. Data are used in Figure 13 of main text.

	Alkyl												Fluoro
n	C1 H's	C2 H's	C3 H's	C4 H's	C5 H's	C6 H's	C7 H's	C8 H's	C9 H's	C10 H's	C11 H's	C12 H's	C1 & C2 H's
4	0.0071	0.0170	0.0304	0.1126	-	-	-	-	-	-	-	-	0.0075
6	0.0074	0.0161	0.0194	0.0376	0.0619	0.1740	-	-	-	-	-	-	0.0055
8	0.0059	0.0132	0.0142	0.0283	0.0327	0.0501	0.0787	0.2021	-	-	-	-	0.0046
12	0.0047	0.0112	0.0103	0.0211	0.0216	0.0339	0.0352	0.0470	0.0496	0.0665	0.0943	0.2287	0.0027

Table S16: Fraction of solvent accessible area of hydrogen atoms by chain position for [$C_n$mim]$_{0.75}$[C_8mimF_{13}]$_{0.25}$[NTf_2]. Data are used in Figure 13 of main text.

N	C1 H's	C2 H's	C3 H's	C4 H's	C5 H's	C6 H's	C7 H's	C8 H's	C9 H's	C10 H's	C11 H's	C12 H's
4	0.357	0.325	0.307	0.312	-	-	-	-	-	-	-	-
6	0.514	0.456	0.485	0.478	0.486	0.484	-	-	-	-	-	-

8	0.574	0.499	0.537	0.541	0.555	0.546	0.592	0.599	-	-	-	-
12	0.840	0.897	0.786	0.845	0.737	0.812	0.742	0.779	0.727	0.745	0.719	0.719

Table S17: Ratio of $[C_n mim]_{(0.75)}[C_8 mimF_{13}]_{0.25}[NTf_2]$ to $[C_n mim][NTf_2]$ solvent-accessible area of hydrogen atoms by chain position. Data are used in Figure 13 of main text.

X	C1 H's	C2 H's	C3 H's	C4 H's	C5 H's	C6 H's	C7 H's	C8 H's
0.00	1.000	1.000	1.000	1.000	1.000	1.000	1.000	1.000
0.10	0.792	0.722	0.745	0.726	0.742	0.756	0.779	0.794
0.25	0.574	0.499	0.537	0.541	0.555	0.546	0.592	0.599
0.50	0.315	0.283	0.321	0.276	0.279	0.276	0.292	0.299
0.75	0.165	0.147	0.129	0.126	0.114	0.120	0.119	0.124
1.00	0.000	0.000	0.000	0.000	0.000	0.000	0.000	0.000

Table S18: Ratio of $[C_8 mim]_{(1-x)}[C_8 mimF_{13}]_x[NTf_2]$ to $[C_8 mim][NTf_2]$ solvent-accessible area of hydrogen atoms by chain position.

3. Surface tension measurements

3.1. Surface tension results $[C_8mim]_{(1-x)}[C_8mimF_{13}]_x[NTf_2]$

x	Surface Tension / mN m^{-1}	1σ SEM	95% CL
0.00	30.42	0.09	0.20
0.05	28.65	0.01	0.03
0.2	25.59	0.08	0.18
0.35	24.30	0.02	0.05
0.5	23.52	0.04	0.10
0.65	22.96	0.04	0.10
0.8	22.52	0.04	0.10
0.95	21.96	0.01	0.03
1.00	21.90	0.02	0.05

Table S19: Surface tension results from $[C_8mim]_{(1-x)}[C_8mimF_{13}]_x[NTf_2]$ for varying bulk mole fraction, *x*.

4. RAS-LIF OH appearance profiles

4.1. Number density profiles for $[C_6mim]_{(1-x)}[C_8mimF_{13}]_x[NTf_2]$ and $[C_4mim]_{(1-x)}[C_8mimF_{13}]_x[NTf_2]$

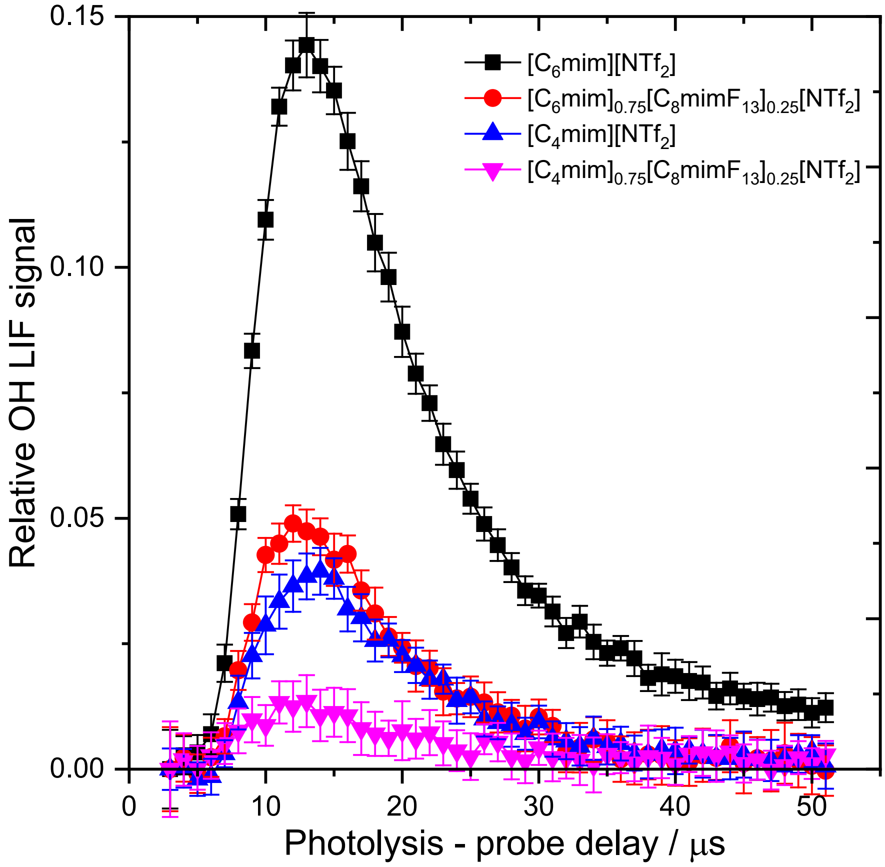

Figure S9: RAS-LIF appearance profiles from $[C_6mim]_{(1-x)}[C_8mimF_{13}]_x[NTf_2]$ and $[C_4mim]_{(1-x)}[C_8mimF_{13}]_x[NTf_2]$ for bulk mole fractions of x = 0 and 0.25. Error bars are 95% CL.

Figure S9 shows the OH density profiles for the C4 and C6 chain length mixtures, $[C_nmim]_{(1-x)}[C_8mimF_{13}]_x[NTf_2]$, with x = 0 and x = 0.25 recorded on the $Q_1(1)$ line. The profiles have very similar peak arrival times, therefore a density to flux transform was not used in the analysis.

5. RAS-LIF rotational distributions

Details of the rotational analysis for the $[C_8mim]_{(1-x)}[C_8mimF_{13}]_x[NTf_2]$ series of mixtures have been given previously.[2] However, it is almost identical to the procedure described here for $[C_{12}mim]_{(1-x)}[C8mimF_{13}]_x[NTf_2]$.

5.1. $[C_{12}mim]_{0.5}[C_8mimF_{13}]_{0.5}[NTf_2]$ and $[C_{12}mim][NTf_2]$ rotationally resolved spectra

Rotationally resolved OH (A-X) (1,0) LIF excitation spectra were recorded from $[C_{12}mim]_{(1-x)}[C_8mimF_{13}]_x[NTf_2]$ for $x = 0$ and $x = 0.5$. The wavelength step size was 0.0020 nm and data was collected for 40 laser shots at each step. The photolysis-probe delay was fixed at 13 µs corresponding to the peak of the appearance profile. For $x = 0$, seven wavelength scans were recoded. For $x = 0.5$, since there was less OH signal from this mixture, twelve scans were recorded. Spectra were recorded on the Q_1 branch only up to $N = 5$, where N is the sum of the electron orbital quantum and rotational quantum numbers.

5.2. Rotational analysis

LIFBASE[3] was used the fit the spectra for both liquids, the resultant populations were normalized over the five rotational levels recorded and averaged over the number of scans taken for each mixture. Figure S10 shows the normalized Q_1 branch populations of OH reactively scattered from $[C_{12}mim]_{(1-x)}[C_8mimF_{13}]_x[NTf_2]$.

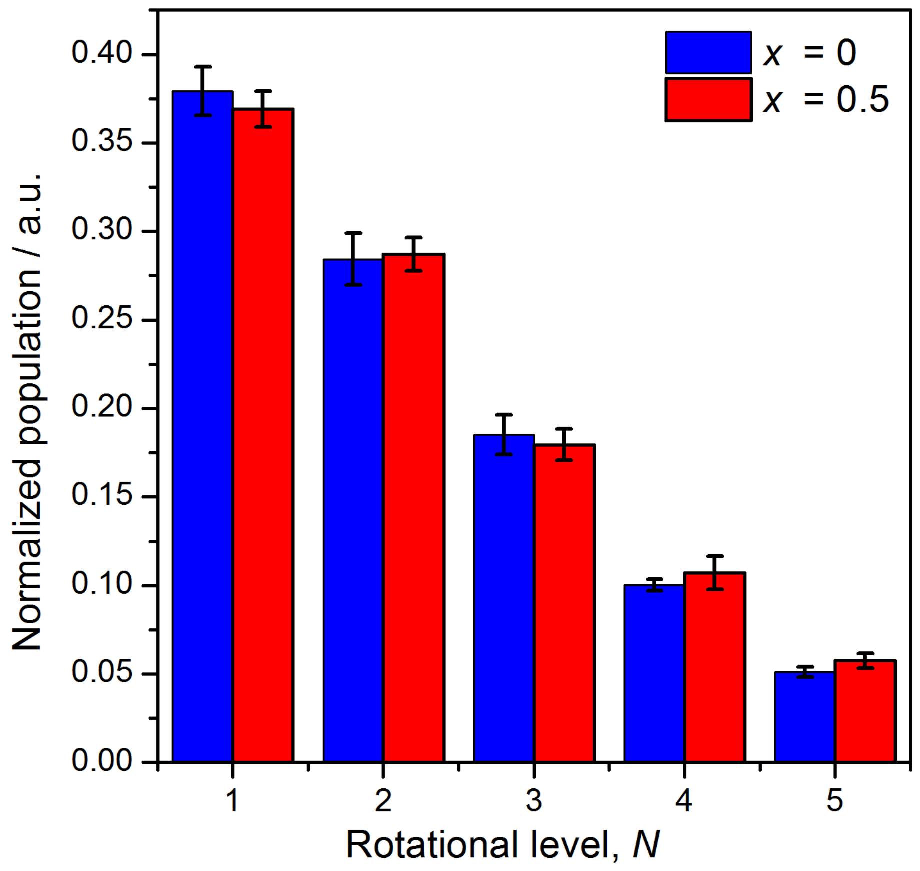

Figure S10: Normalised OH rotational populations from $[C_{12}mim][NTf_2]$ (*x* = 1.0) and with bulk mole fraction, *x* = 0.5, corresponding to $[C_{12}mim]_{0.5}[C_8mimF_{13}]_{0.5}[NTf_2]$. Error bars are 95% CL.

6. Integrated RAS-LIF OH number densities to derive reactivity

The OH densities for all liquids were integrated to create a value called reactivity, a single numerical value that represents the exposure of the alkyl chains to incident $O(^3P)$ atoms at the liquid surface. This was done by integrating the OH density as a function of time between 6 μs and 30 μs with a 1 μs step size. The reactivities of each liquid were divided by the reactivity of squalane, to create a dimensionless relative reactivity. This process helped to reduce systematic error due to variations in the experimental conditions.

To help estimate and reduce the experimental uncertainty, the C8 and C12 pure liquids, $[C_{8,12}mim][NTf_2]$, were measured at the start and end of the experiment. For the C8 system, after all compositions had been measured, fresh samples of $[C_8mim][NTf_2]$ (a commercial sample, IoLiTec, and a sample synthesised as part of this work) were loaded into a separate liquid baths, and their reactivates measured. For the C12 system, only a commercial sample (IoLiTec) was measured at the end of the experiments, after mixtures has been measured. The reactivities for $[C_{8,12}mim][NTf_2]$ were calculated using a weighted average with instrumental weighting, $1/\sigma^2$. This averaged value was then taken as the reactivity of the pure component. For the C4 and C6 chain lengths only a single mixture was studied, x = 0.25.

6.1. $[C_{12}mim]_{(1-x)}[C_8mimF_{13}]_x[NTf_2]$ mixtures

x	Relative Reactivity	1σ SEM	Normalized reactivity	1σ SEM	95% CL
0.000	0.625	0.002	1.000	0.006	0.000
0.020	0.557	0.003	0.891	0.006	0.013
0.050	0.438	0.003	0.701	0.006	0.013
0.100	0.334	0.002	0.534	0.004	0.008
0.250	0.211	0.001	0.338	0.002	0.005
0.370	0.161	0.001	0.258	0.002	0.005
0.500	0.122	0.001	0.195	0.002	0.005
0.750	0.074	0.001	0.118	0.001	0.003

Table S20: Relative and normalized reactivities for $[C_{12}mim]_{(1-x)}[C_8mimF_{13}]_x[NTf_2]$ mixtures with varying x.

Measurement	Relative Reactivity	1σ SEM
1	0.632	0.003
Commercial Sample	0.611	0.004
Weighted Average	0.625	0.002

Table S21: Confirmatory measurements of relative reactivity for custom-synthesized and commercial samples of [C_{12}mim][NTf_2] and resulting weighted average.

6.2. [C_8mim]$_{(1-x)}$[C_8mimF_{13}]$_x$[NTf_2] mixtures

Previously published results[2] show tabulated values for reactivities relative to squalane with bulk mole fraction x, for [C_8mim]$_{(1-x)}$[C_8mimF_{13}]$_x$[NTf_2] mixtures, the data are repeated here for convenience and include the normalized reactivity and associated errors.

x	Relative Reactivity	1σ SEM	Normalized Reactivity	1σ SEM	95% CL
0.000	0.338	0.001	1.000	0.006	0.000
0.050	0.226	0.002	0.669	0.007	0.018
0.100	0.183	0.002	0.543	0.006	0.013
0.250	0.125	0.001	0.370	0.005	0.010
0.370	0.086	0.002	0.256	0.005	0.011
0.500	0.073	0.006	0.218	0.017	0.038
0.750	0.044	0.001	0.132	0.004	0.008
0.830	0.041	0.001	0.122	0.004	0.009
1.000	-0.007	0.006	-0.020	0.018	0.042

Table S22: Relative and normalized reactivities for [C_{12}mim]$_{(1-x)}$[C_8mimF_{13}]$_x$[NTf_2] mixtures with varying x.

Measurement	OH Density	1σ SEM
1	0.3196	0.0065
2	0.3170	0.0022
Commercial Sample	0.3555	0.0020
Weighted Average	0.3376	0.0014

Table S23: Confirmatory measurements of relative reactivity for custom-synthesized and commercial samples of [C_8mim][NTf_2] and resulting weighted average.

6.3. [C_6mim]$_{(1-x)}$[C_8mimF_{13}]$_x$[NTf_2]

The C6 liquids were studied by having the pure liquid, [C_6mim][NTf_2] and the x = 0.25 mixture in the carousel at the same time.

X	Relative Reactivity	1σ SEM	Normalized Reactivity	1σ SEM	95% CL
0.00	0.143	0.001	1.000	0.007	0.000
0.25	0.044	0.001	0.310	0.005	0.011

Table S24: Relative and normalized reactivities for [C_6mim]$_{(1-x)}$[C_8mimF_{13}]$_x$[NTf_2] mixtures with varying x.

6.4. [C_4mim]$_{(1-x)}$[C_8mimF_{13}]$_x$[NTf_2]

The C4 liquids were studied by having the pure liquid, [C_4mim][NTf_2] and the x = 0.25 mixture loaded in the carousel at the same time.

x	Relative Reactivity	1σ SEM	Normalized Reactivity	1σ SEM	95% CL
0.000	0.036	0.001	1.000	0.025	0.000
0.25	0.012	0.001	0.347	0.021	0.046

Table S25: Relative and normalized reactivities for [C_4mim]$_{(1-x)}$[C_8mimF_{13}]$_x$[NTf_2] mixtures with varying x.

7. Supplementary references

1. Leach, A. R., *Molecular Modelling, Principles and Applications*. 2nd ed.; Prentice Hall: Harlow, England, 2001.

2. Smoll, E. J.; Tesa-Serrate, M. A.; Purcell, S. M.; D'Andrea, L.; Bruce, D. W.; Slattery, J. M.; Costen, M. L.; Minton, T. K.; McKendrick, K. G., Determining the composition of the vacuum-liquid interface in ionic-liquid mixtures. *Faraday Discussions* **2018,** *206*, 497-522.

3. Luque, J.; Crosley, D. R. *LIFBASE: Database and spectral simulation (version 1.5)*, SRI International Report MP 99-009: 1999.
